# Vision-Based Detection of Estrus-Related Interaction Behavior in Hanwoo Cattle Using Lightweight YOLO Models

**DOI:** 10.3390/ani16193054

**Published:** 2026-09-28

**Authors:** Usama Hanif, Dae-Yeong Kang, Mohammad Ismail, Pirlepesov Daniyar Maratovich, Seong-hun Kim, Hyeon-Tae Kim

**Affiliations:** 1 Department of Bio-Systems Engineering, Gyeongsang National University, Jinju 52828, Republic of Korea; usama@gnu.ac.kr (U.H.); ismail@gnu.ac.kr (M.I.); 2 Department of Smart Farm, Gyeongsang National University, Jinju 52828, Republic of Korea; dykang@gnu.ac.kr (D.-Y.K.); daniyar@gnu.ac.kr (P.D.M.); tjdgnszhd@naver.com (S.-h.K.); 3 Department of Bio-Industrial Machinery Engineering, Institute of Smart Space Agriculture (ISSA), Gyeongsang National University, Jinju 52828, Republic of Korea

**Keywords:** Hanwoo, estrus detection, object detection, annotation strategy, data augmentation, precision livestock farming

## Abstract

Timely estrus detection determines reproductive efficiency in Hanwoo cattle, yet visual observation is increasingly difficult as herd sizes grow and standing behavior becomes less consistently expressed. Fixed farm cameras offer a non-invasive alternative, but mounting, chin-resting and sniffing involve two overlapping animals and occur rarely, complicating automated detection. This study evaluated how annotation strategy, augmentation policy and detector choice affect detection of these cues. A surveillance dataset of 846 red–green–blue (RGB) frames, containing 200 estrus-interaction instances against 2180 posture instances, was recorded at a commercial farm. Three annotation strategies, partial bounding-box label (PBL), full single bounding-box label (FSBL) and full dual bounding-box label (FDBL), were combined with three augmentation stages and three lightweight detectors (YOLOv5n, YOLOv8n, YOLO11n), giving 27 configurations. Rare-class performance varied approximately twice as widely as overall performance and depended more on annotation and augmentation than on detector family. PBL maximized sensitivity (recall 0.718), FSBL maximized localization quality (average precision, AP50 0.798) and overall accuracy (mean average precision, mAP50 0.864), and FDBL preserved relational context (recall 0.700, AP50 0.775). Annotation and augmentation should be selected according to whether the purpose is screening candidate interactions or supporting expert confirmation. Detections represent a behavioral proxy, not physiological confirmation of estrus.

## Simple Summary

Knowing when a cow is ready to breed is one of the most important daily judgments on a beef farm. If the moment is missed, the cow is not bred on time, the gap between calves grows longer, and the farm earns less. This has become harder on Korean native cattle farms, because herds are larger, fewer people are available to watch the animals, and the clearest visual sign is no longer shown by every cow in heat. Many farms already have cameras in their barns. This study asked whether those cameras could help by automatically spotting the brief physical contacts between cows that happen around breeding time. Twenty-seven different ways of building such a system were tested on the same set of farm images. Most usefully, how the behavior is marked out in the training images and how those images are varied during training mattered more than which detection program was used. Farms that want to catch as many possible events as they can should mark the images one way, while those needing precise positions for a specialist to check should mark them another way. What the cameras find is a sign worth checking, not a confirmed diagnosis.

## 1. Introduction

Reproductive efficiency in Hanwoo production depends on identifying estrus accurately and acting on it within a narrow window. Missed or mistimed estrus lengthens calving intervals and raises the production cost of each calf. Changes in the structure of Korean beef production have made this harder rather than easier. Increases in herd size and reductions in available labor have reduced the time available for individual animal observation, and reproductive efficiency has fallen accordingly [1].

The behavioral basis of visual detection has also weakened. Standing to be mounted is recognized as the primary behavioral sign of estrus, but its expression has declined substantially in modern dairy cattle, from approximately 80% to 50% of estrous episodes, together with a shortening of standing duration from around 15 h to fewer than eight [2,3,4]. Comparable difficulties are reported in Hanwoo, where visual observation confirms only 50% to 60% of estrous events relative to biosensor-supported detection, and where detection is least reliable at night when behavioral cues are harder to see and routine inspection is not performed [5]. Secondary signs such as mounting attempts, chin-resting and sniffing therefore carry proportionally more of the observational burden than they once did.

Sensor-based systems have been developed in response. Neck-collar accelerometers, pedometers and ruminoreticular biocapsules detect estrus-associated changes in activity or physiology with useful accuracy [5,6]. Each requires a device on every animal, however, together with fitting, periodic adjustment and maintenance, and these requirements scale directly with herd size [6]. Non-contact alternatives have also been examined. Infrared thermography has been proposed as a non-invasive route to estrus detection in cattle and buffalo [7], and a thermal tracking system applied to Hanwoo detected a 2.35-fold increase in movement and an 11% reduction in feed intake on the day of estrus [8]. These approaches infer estrus from aggregate activity or physiological change rather than from the interactions themselves.

Fixed red–green–blue (RGB) cameras occupy a different position. Many commercial Hanwoo farms already operate surveillance cameras for security and general supervision, so pen-level observation is possible without additional per-animal hardware [9,10]. Near-animal sensing of this kind can complement wearable devices by enabling non-contact monitoring that scales to herd level [11]. Camera-based estrus detection has been demonstrated: background subtraction of whole-animal movement identified estrus-associated activity increases in tie-stalled cows, using one camera view per animal and no deep-learning detector [12]. Group housing removes that simplifying condition, because occlusion in crowded pens and restricted movement reduce the visibility of behaviors such as mounting, and camera placement and barn layout become determinants of performance [11].

Deep learning has been applied widely to cattle imagery but predominantly to behaviors of individual animals. Multi-view action recognition has distinguished standing, walking, resting, ruminating and feeding in closed barns [13], and lightweight detectors have recognized posture and daily behavior in beef cattle under varied weather [14]. Interaction behaviors differ from these in kind rather than degree. A posture belongs to one animal and can be enclosed by one box, whereas mounting, chin-resting and sniffing involve at least two animals in a specific spatial relationship. The participating bodies overlap, the two animals occupy different roles, and the visual evidence for the behavior lies partly in the geometry between them rather than in either animal alone. Consequently, the spatial extent of the target is not self-evident: the same event can legitimately be represented by the contact region, by the interacting animal, or by both participants together.

Work on Hanwoo specifically has concentrated on identity rather than behavior. Individual animals have been identified from muzzle pattern images using deep learning [15], and re-identification across viewpoints has been addressed for this weakly textured breed [16], which supports long-term monitoring of individual animals. Machine learning has also been applied to Hanwoo production records for body weight prediction [17]. These studies establish that useful visual representations can be extracted from Hanwoo imagery, but their targets are identity and production traits rather than reproductive interaction.

Two further considerations shape how such a system should be designed. Estrus-associated interactions are sparse relative to routine posture: in the present dataset, they appear in 197 of 788 labeled images, so the detector must learn from a small number of positive examples. The intended use also differs by farm. Screening aims to capture as many candidate interactions as possible for subsequent human review and tolerates false positives in exchange for sensitivity. Confirmation support aims to produce spatially accurate detections that an operator or veterinarian can interpret, and values localization quality over raw sensitivity. This distinction follows the recommendation that sensor interpretation be tied explicitly to the intended management decision [11].

To our knowledge, mounting, chin-resting and sniffing have not been reported as labeled object-detection classes for RGB surveillance of group-housed Hanwoo cattle. Among the cattle-vision studies examined here, neither spatial annotation strategy nor augmentation composition has been evaluated as a controlled experimental variable for a sparsely represented interaction class [12,13,14,15,16]. The unresolved question is therefore not whether computer vision can recognize cattle behavior but how a detection system should be formulated when the target is a rare, spatially relational reproductive interaction. Accordingly, this study evaluated three annotation strategies, three augmentation stages and three lightweight detector families as co-equal design variables on a fixed Hanwoo dataset, using an identical data partition and training schedule across all 27 configurations. The specific aims were to determine how spatial annotation affects the proportion of labeled interactions detected, how augmentation composition affects learnability of the rare class, and which configurations best serve screening and confirmation support, respectively.

## 2. Materials and Methods

### 2.1. Ethical Statement

The data comprised RGB video frames of Hanwoo cattle recorded non-invasively from fixed surveillance cameras at a commercial farm under routine management, together with publicly available frames from a national open data platform. All recordings were obtained under normal husbandry conditions without alteration of routine management. Because the study involved only non-invasive observation, with no experimental procedure, handling or intervention performed on the animals, approval by an Institutional Animal Care and Use Committee was not required by the institution.

### 2.2. Study Design

**Figure 1:**
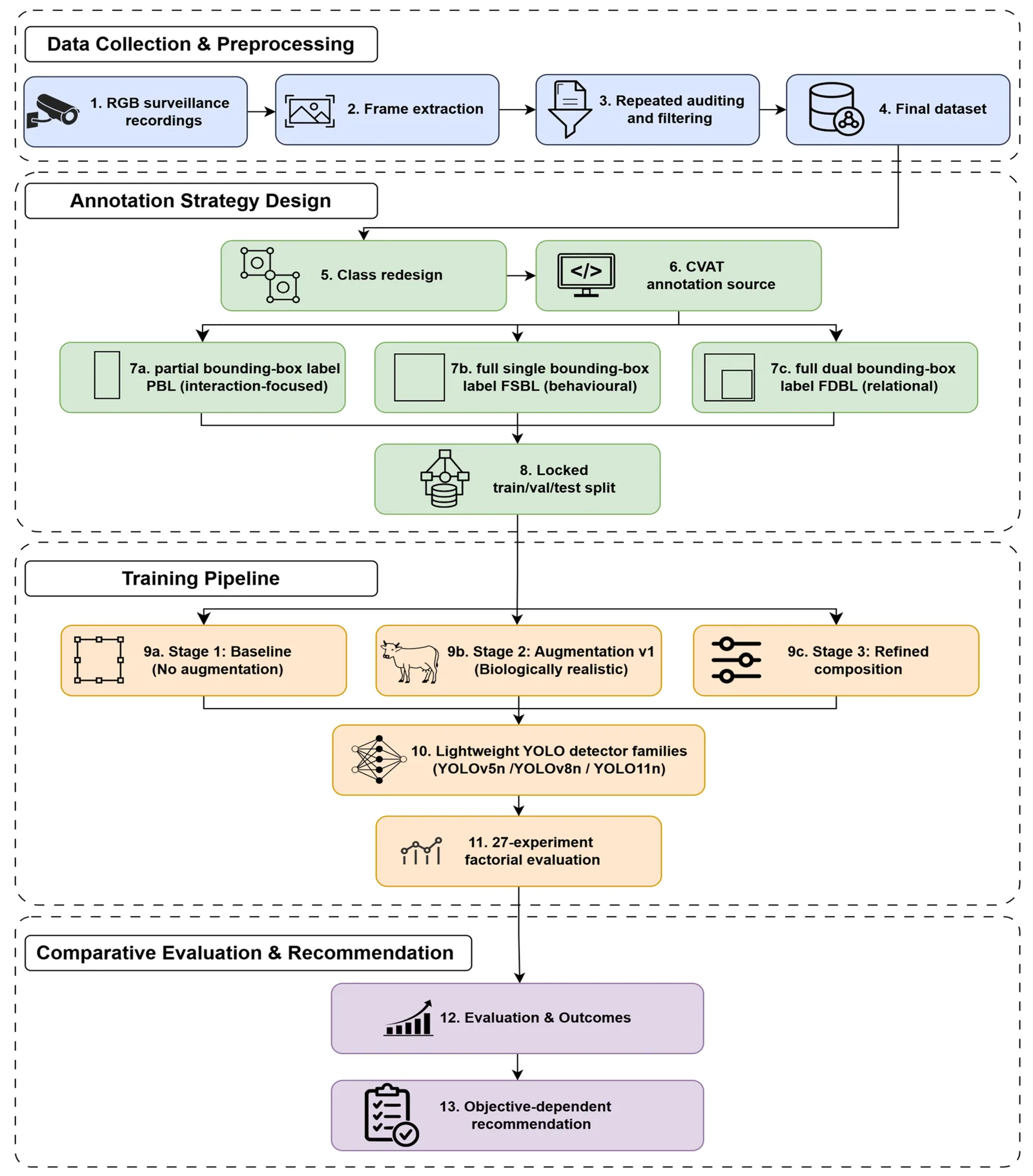
Overall workflow of the controlled evaluation, from RGB frame curation and XML-based annotation to the 27-run annotation, augmentation and detector matrix. RGB, red–green–blue; XML, Extensible Markup Language.

The study was designed as a controlled comparative evaluation of lightweight object detectors for frame-level detection of estrus-related interaction behavior in Hanwoo cattle (Figure 1). Three factors were varied in a complete factorial structure: annotation strategy at three levels, augmentation stage at three levels, and detector family at three levels, giving 27 configurations. All configurations used the same dataset partition, image resolution, training schedule and optimization defaults, so that differences in performance could be attributed to the experimental factors rather than to data partitioning or model-specific tuning. The analytical scope was limited to frame-level detection of visually defined behaviors in RGB images and did not extend to physiological estrus diagnosis, sensor fusion, temporal sequence modeling or cross-farm validation.

### 2.3. Data Acquisition and Curation

**Figure 2:**
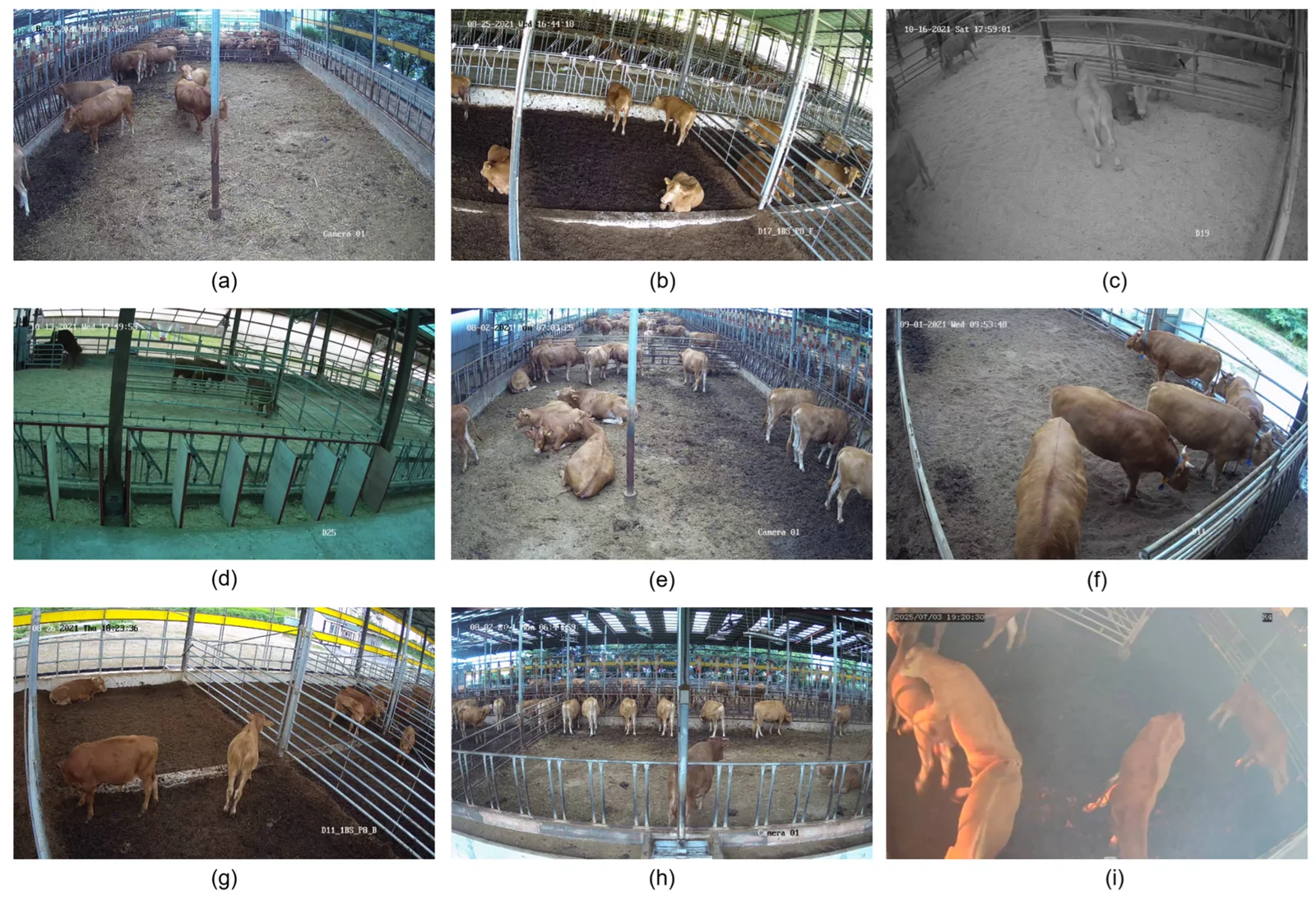
Variation in recording conditions across the dataset. Panels (**a**–**h**) show frames recorded at the study farm between August and November 2021, differing in camera viewpoint, illumination, pen structure, flooring, animal density and animal distance from the camera, including infrared night-time recording in panel (**c**). Panel (**i**) shows a representative frame from the public dataset.

RGB surveillance video was recorded at Samtteul Ranch, a commercial Hanwoo farm in Icheon-si, Gyeonggi-do, Republic of Korea. Recordings were obtained from multiple group-housed pens between 2021 and 2025 using fixed-position cameras with 2.8 mm wide-angle lenses (VIGI C340I; TP-Link, Shenzhen, China) mounted overhead on the barn structure at an estimated height of approximately 3 m above the pen floor, inferred by comparison with known structural dimensions of the pen railings and feed barrier. The cameras provided an oblique downward view covering the pen floor, and mounting position and viewing angle differed between pens. Because recording spanned multiple pens and seasons, the footage captured substantial variation in illumination, animal density, pen structure, flooring and animal distance from the camera, including infrared night-time recording (Figure 2). Across the sampled frames, between four and fifteen animals were visible within the monitored pen area, depending on pen and time of day.

Additional Hanwoo frames were obtained from the public AI-Hub livestock datasets to increase the number of rare interaction cases available for training. Both sources also contained dairy cattle, and only Hanwoo frames were retained so that the final dataset was breed-specific. The privately collected farm-surveillance images are not publicly available because they were recorded under institutional and laboratory data-use restrictions. The public frames are available from the AI-Hub platform subject to that platform’s access conditions and licensing terms.

Approximately 2500 still frames were extracted from the recorded video. These were audited repeatedly to remove non-target images, severely blurred or low-resolution frames, scenes without visible animals, dairy-cattle frames, and interaction events that could not be interpreted unambiguously. Candidate frames were selected to capture estrus-interaction events together with the periods immediately preceding and following them, and to include standing and lying frames so that postures could be distinguished from interactions. This yielded 734 curated Hanwoo frames, to which 112 public frames were added.

The locked dataset comprised 846 RGB frames, of which 788 were labeled and 58 were background-only images containing no target animal. Among the labeled images, 197 contained at least one estrus-interaction instance, confirming a strongly imbalanced rare-event setting. The dataset was partitioned once at frame level into training, validation and test subsets in a 593/128/125 split, and this partition was held constant across all 27 configurations (Table 1). Within the training subset, 552 images were labeled and 41 were background-only; the validation subset contained 119 labeled and 9 background-only images; and the test subset contained 117 labeled and 8 background-only images. Because the partition was applied at frame level, reported performance describes within-dataset detection under matched conditions across all configurations.

### 2.4. Class Formulation

Three frame-level classes were defined: standing, lying and estrus-interaction. Standing and lying served as posture reference classes, occurring frequently and providing stable non-interaction categories against which the detector could learn to discriminate. Estrus-interaction was defined as a unified rare visual class encompassing mounting, chin-resting and sniffing. Mounting was treated as the most explicit visually observable reproductive interaction, while chin-resting and sniffing were included as associated cues that may precede or accompany mounting within the same observation period.

**Figure 3:**
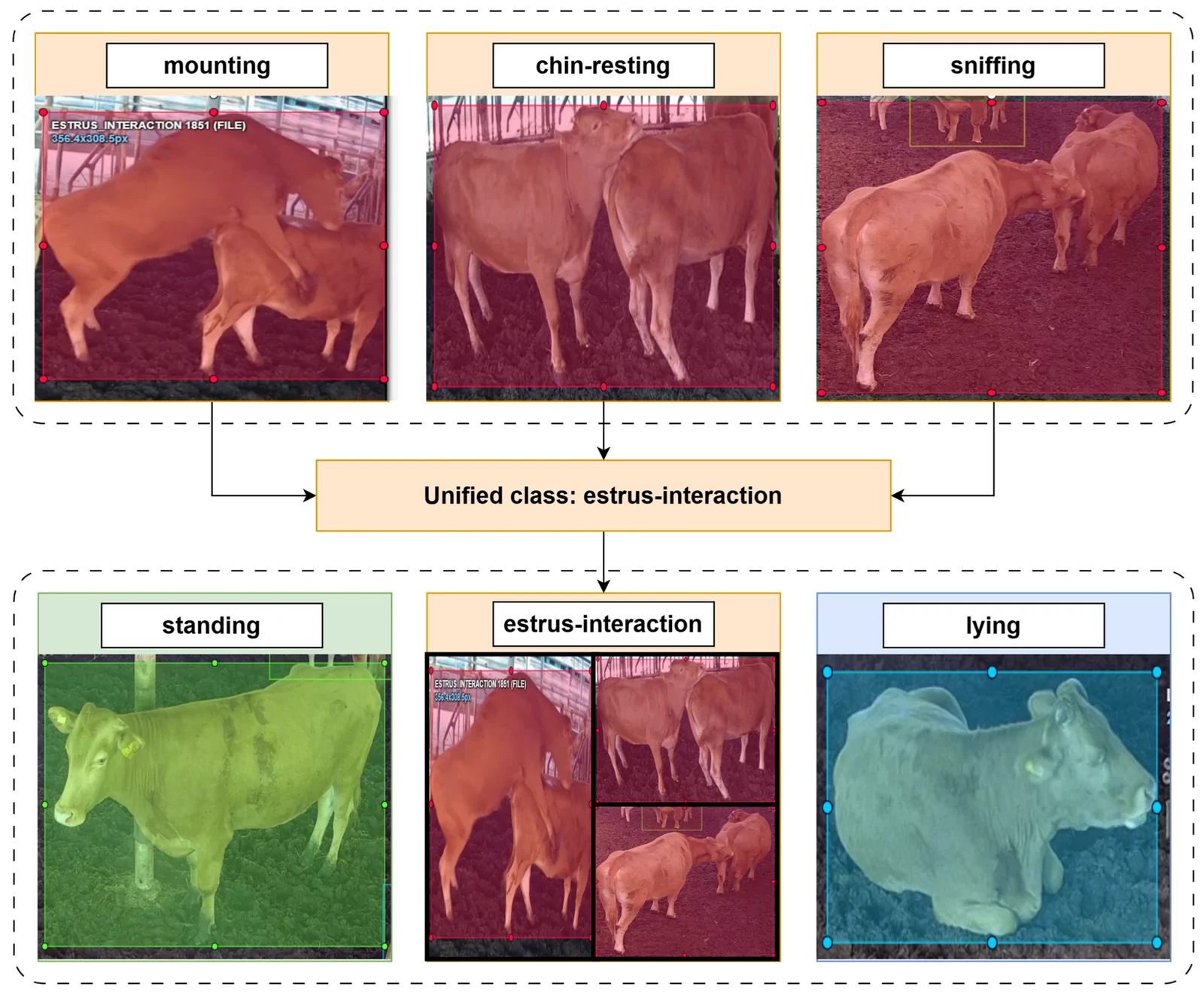
Unified formulation of the estrus-interaction class from mounting, chin-resting and sniffing, with standing and lying as posture reference classes. Shaded boxes show the annotated estrus-interaction (red), standing (green) and lying (blue) regions; other overlaid boxes and text are displayed by the annotation tool.

This formulation followed established descriptions of estrus-associated behavioral signs in cattle and Hanwoo-specific reports of visual estrus behavior [3,18,19,20], and it is supported by a beef-cattle annotation precedent in which chin-resting and sniffing were labeled alongside mounting as co-occurring cues [21]. The class is a behavior-level visual proxy for estrus-related interaction. It does not imply hormonal validation, animal intent, standing heat, or sequence-based confirmation of estrus status. The unified class formulation is illustrated in Figure 3, and representative frames in which chin-resting or sniffing occurred near mounting within the same camera scene are provided in Figure S1.

### 2.5. Annotation Strategies

An interaction involves at least two animals in a specific spatial relationship, and the same event can therefore be represented in more than one legitimate way. Three annotation strategies were defined to test how bounding-box formulation changes the visual evidence available to the detector, and each was applied consistently to the same underlying frames. This design follows object-detection studies showing that annotation quality, bounding-box geometry and spatial label representation influence model training and evaluation, particularly when the target class is rare or visually ambiguous [22,23,24,25].

#### 2.5.1. Partial Bounding-Box Label

The partial bounding-box label (PBL) used a compact, event-focused representation in which a tight box was placed around the visually salient interaction zone. This strategy was intended to minimize background burden and concentrate supervision on the localized contact region. Its expected advantage was increased sensitivity to subtle interaction cues; its expected limitation was reduced body-level and contextual information.

#### 2.5.2. Full Single Bounding-Box Label

The full single bounding-box label (FSBL) enclosed the participating interaction region within one inclusive behavioral box. This preserved more body-level structure than PBL while maintaining a single target label. Its purpose was to test whether a broader spatial representation improved localization quality and overall detection stability for the rare class.

#### 2.5.3. Full Dual Bounding-Box Label

The full dual bounding-box label (FDBL) used a relational representation in which the estrus-interaction box was paired with a second box marking the recipient cow, defined as the animal receiving the mounting, sniffing or chin-resting interaction. FDBL therefore used four annotation classes, whereas PBL and FSBL used three. The recipient-cow label was included because standing to be mounted is widely treated as a primary behavioral sign of estrus, but it was used here only as a frame-level relational annotation and should not be interpreted as physiological confirmation of estrus status. This strategy is an annotation-representation design within a frame-level object-detection study, not a recipient-cow identification, tracking or temporal interaction model [26,27].

**Figure 4:**
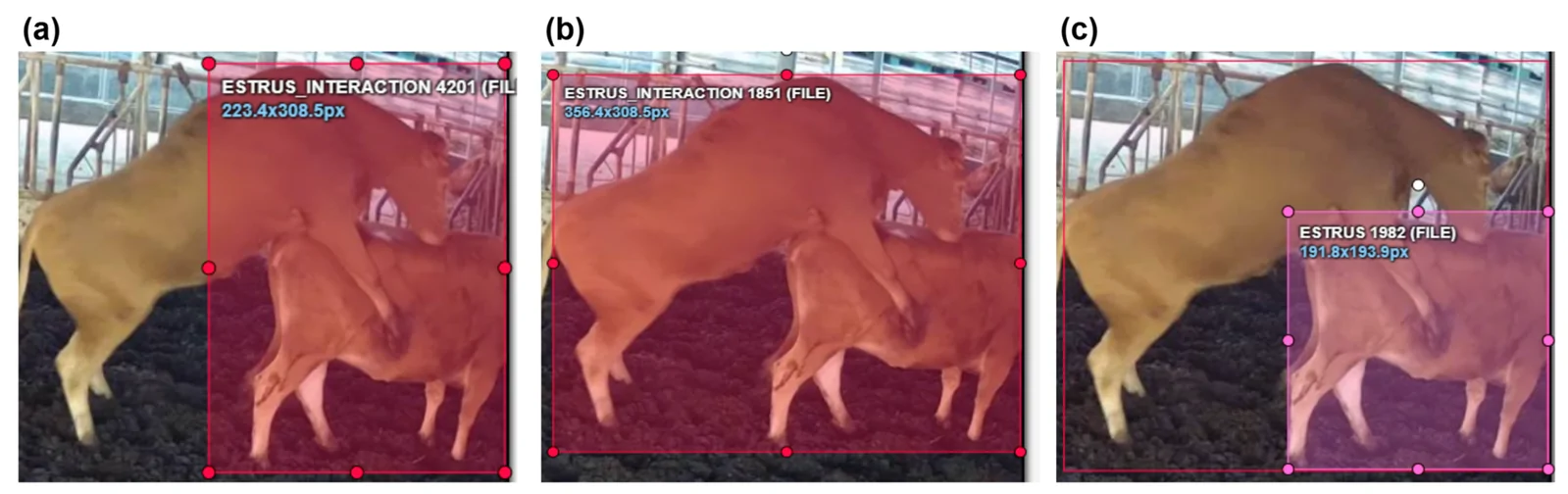
Annotation-strategy examples. (**a**) PBL, compact interaction-zone box; (**b**) FSBL, inclusive single box; (**c**) FDBL, estrus-interaction box paired with recipient-cow box. Red boxes mark the estrus-interaction label, and the pink box in (**c**) marks the recipient-cow label; overlaid text is displayed by the annotation tool.

Together, PBL emphasized the local interaction signal, FSBL emphasized inclusive behavioral localization, and FDBL emphasized relational context. Definitions, box composition and intended role are summarized in Table 2, and representative frame-level examples are shown in Figure 4.

To ensure consistent label geometry and class mapping across all 27 configurations, a single CVAT Extensible Markup Language (XML) master annotation file (CVAT, Computer Vision Annotation Tool; https://www.cvat.ai, accessed on 23 August 2026) was treated as the authoritative annotation source, and all You Only Look Once (YOLO)-format label files were regenerated programmatically from it rather than reused from earlier exports [22,28]. Core class-instance counts were identical across the three strategies, comprising 1590 standing, 590 lying and 200 estrus-interaction bounding boxes. The 200 estrus-interaction instances occurred across 197 images, indicating that a small number of frames contained more than one labeled interaction. PBL and FSBL therefore each contained 2380 boxes, whereas FDBL added 200 recipient-cow boxes for a total of 2580 (Table 3).

### 2.6. Detector Families and Training Configuration

Three lightweight detector families were evaluated: YOLOv5n, YOLOv8n and YOLO11n. These nano-scale models were selected to compare compact detectors from different YOLO generations under identical dataset partitions, augmentation stages and training settings. Lightweight YOLO variants are widely used in agricultural and livestock computer vision because they balance detection accuracy, inference speed, model size and implementation feasibility for real-time monitoring [28,29,30].

**Figure 5:**
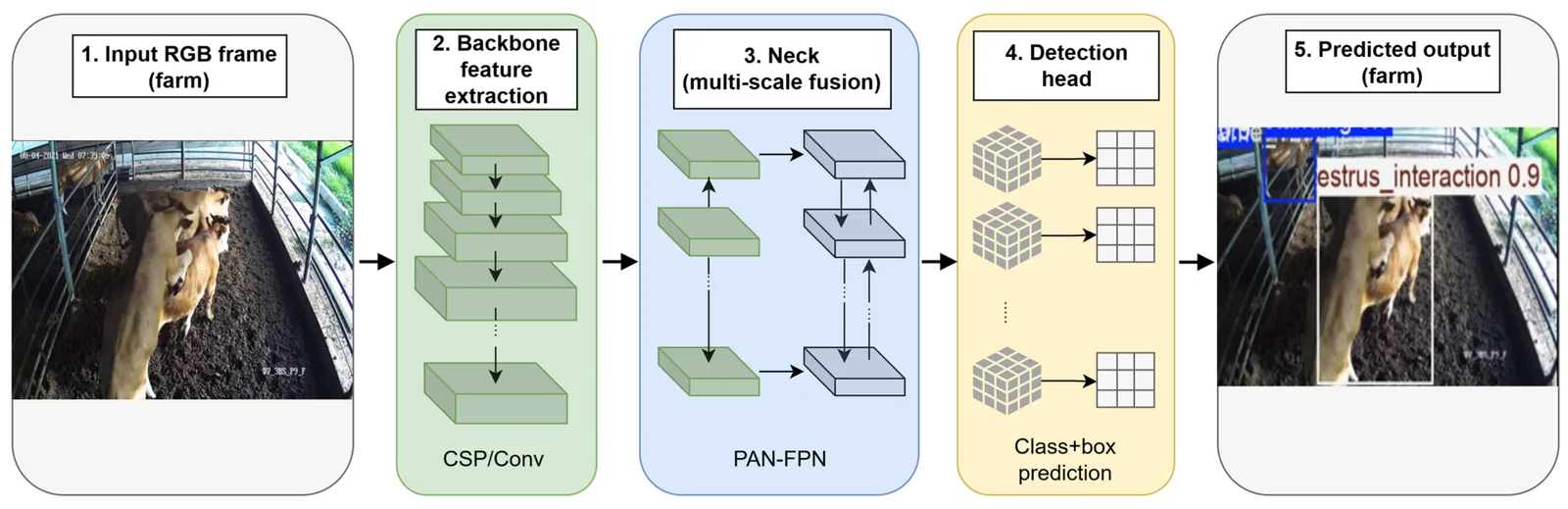
Representative lightweight single-stage detection workflow, comprising input, backbone feature extraction, multi-scale fusion, detection head and prediction. Arrows indicate the direction of data flow, and colors distinguish the processing stages.

The purpose of including three families was not to propose or modify a detector architecture but to provide controlled detection backbones for evaluating how annotation strategy and augmentation stage influence frame-level behavior detection. The three families differ in internal implementation across YOLO generations but share the single-stage workflow of RGB image input, backbone feature extraction, multi-scale feature aggregation, detection-head processing and final class and bounding-box prediction. Figure 5 presents a representative lightweight single-stage detection workflow rather than an exact architecture diagram for any one model.

All families were trained and evaluated under the same locked design. The training, validation and test splits; input image size; batch size; epoch budget; early-stopping patience and augmentation stage were held constant for the corresponding experimental condition. Remaining optimizer and learning-rate schedule settings followed framework defaults for each family. A fixed random seed of 0 was set and deterministic mode was enabled for all runs. Non-maximum suppression used an intersection-over-union threshold of 0.7, and the confidence threshold was left at the framework default. Training used the Ultralytics implementation (version 8.4.30; Ultralytics, Frederick, MD, USA) for the YOLOv8n and YOLO11n experiments and the corresponding YOLOv5 repository implementation (release v7.0) for the YOLOv5n experiments. Models were trained on a workstation with an Intel Core i9-10900KF processor (Intel, Santa Clara, CA, USA), 64 GB RAM and an NVIDIA RTX 2070 graphics card with 8 GB memory (NVIDIA, Santa Clara, CA, USA), using Python 3.11.1, PyTorch 2.7.1 and CUDA 11.8. Custom scripts for annotation regeneration and metric extraction are available from the corresponding author on reasonable request. The shared configuration is given in Table 4.

### 2.7. Augmentation Design

Three augmentation stages were evaluated as a controlled experimental factor rather than as fixed preprocessing. Baseline used no augmentation and established clean detector behavior without synthetic variability. Augmentation v1 applied moderate, biologically realistic transformations to single images, including horizontal flipping, mild translation, moderate scaling and restrained perturbation of hue, saturation and value. Refined composition applied a targeted compositional enrichment policy designed to improve estrus-interaction learnability while preserving behavioral realism.

Transformations that could distort posture or interaction geometry were disabled at every stage. These comprised vertical flipping, rotation, shear, perspective warping, copy-paste and random erasing. Refined composition additionally introduced mosaic at a probability of 0.2 and mixup at 0.1, applied at low probability so that composite images remained infrequent relative to unmodified frames, and mosaic was closed during the final ten epochs so that training concluded on unaltered images. This design is consistent with the requirement for semantically meaningful augmentation in object detection and animal phenotyping [28,31,32]. Complete locked parameter values for each stage are provided in Table S1, and stage policy and rationale are summarized in Table 5.

### 2.8. Evaluation Metrics

Detection performance was evaluated using precision, recall, average precision (AP) and mean average precision (mAP), together with two class-specific metrics calculated for the rare estrus-interaction class. General metrics summarized detector behavior across all labeled classes, and class-specific metrics quantified performance on labeled estrus-interaction instances alone.

A detection was classified as a true positive (TP) when the intersection over union (IoU) between the predicted bounding box and the ground-truth bounding box met or exceeded a defined threshold. Detections below the threshold and duplicate detections of the same ground-truth instance were classified as false positives (FPs). Ground-truth instances that the model failed to detect were classified as false negatives (FNs). IoU was calculated using Equation (1):

(1) $$IoU\;=\;\frac{{Area(B}_{p}\cap B_{g})}{{Area(B}_{p}\cup B_{g})}$$

where *B_p_* is the predicted bounding box and *B_g_* is the ground-truth bounding box. Precision and recall were calculated using Equations (2) and (3):

(2) $$Precision=\frac{TP}{\left(TP+FP\right)}$$

(3) $$Recall=\frac{TP}{\left(TP+FN\right)}$$

AP summarized the precision–recall curve for a single class as the area under that curve, calculated using Equation (4):

(4) $$AP_{i}=\int_{0}^{1}P_{i}\left(R\right)dR$$

where *P_i_*(*R*) denotes precision for class *i* expressed as a function of recall. Mean average precision was obtained by averaging AP across the *C* evaluated classes using Equation (5):

(5) $$mAP=\frac{1}{C}\sum_{i=1}^{C}{AP}_{i}$$

where *C* = 3 for PBL and FSBL, and *C* = 4 for FDBL, which additionally includes the recipient-cow class. Two mAP variants were reported. mAP50 is the mean AP computed at a single IoU threshold of 0.50 and reflects general detection quality at a permissive localization threshold. mAP50–95 was calculated by averaging mAP across ten IoU thresholds from 0.50 to 0.95 in steps of 0.05, as shown in Equation (6):

(6) $${mAP}_{50-95}=\frac{1}{10}\sum_{t}{mAP}_{t}$$

where *t* denotes an IoU threshold in the set {0.50, 0.55, …, 0.95} and mAP*_t_* denotes the mAP computed at that threshold.

Because the dataset exhibited a strongly imbalanced, long-tailed distribution in which estrus-interaction was the rare target class, two class-specific metrics were reported alongside the general metrics. Estrus-interaction (EI) Recall was recall computed for the estrus-interaction class alone, quantifying rare-class sensitivity. EI AP50 was AP computed at an IoU threshold of 0.50 for that class alone, quantifying rare-class detection and localization quality. Class-specific metrics were prioritized because overall mAP50 can be dominated by the more frequent standing and lying classes. Because FDBL used an expanded label space, overall mAP values for FDBL were interpreted within that four-class configuration, and EI Recall and EI AP50 were used for all cross-strategy comparisons.

### 2.9. Statistical Analysis

No inferential statistical analyses were performed for the complete 27-configuration comparative evaluation, because each configuration was trained once using a fixed training, validation and test partition; these values represent comparative performance under controlled experimental conditions rather than statistically estimated differences between configurations. To quantify run-to-run variability, the three best-performing configurations identified from this matrix were additionally retrained with four further random seeds (five seeds total) and are reported as mean ± standard deviation (SD) alongside the original single-seed values (Section 3.11).

## 3. Results

### 3.1. Dataset Composition

The locked dataset comprised 846 RGB frames, of which 788 were labeled and 58 contained no target animal. Estrus-interaction appeared in 197 of the 788 labeled images, giving 200 instances in total, against 1590 standing and 590 lying instances. The rare class therefore represented 8.4% of all annotated bounding boxes under PBL and FSBL, and 7.8% under FDBL, which added 200 recipient-cow boxes. Recording conditions varied across pens and months, with between four and fifteen animals visible in the monitored area.

### 3.2. Overall Detection Performance

**Figure 6:**
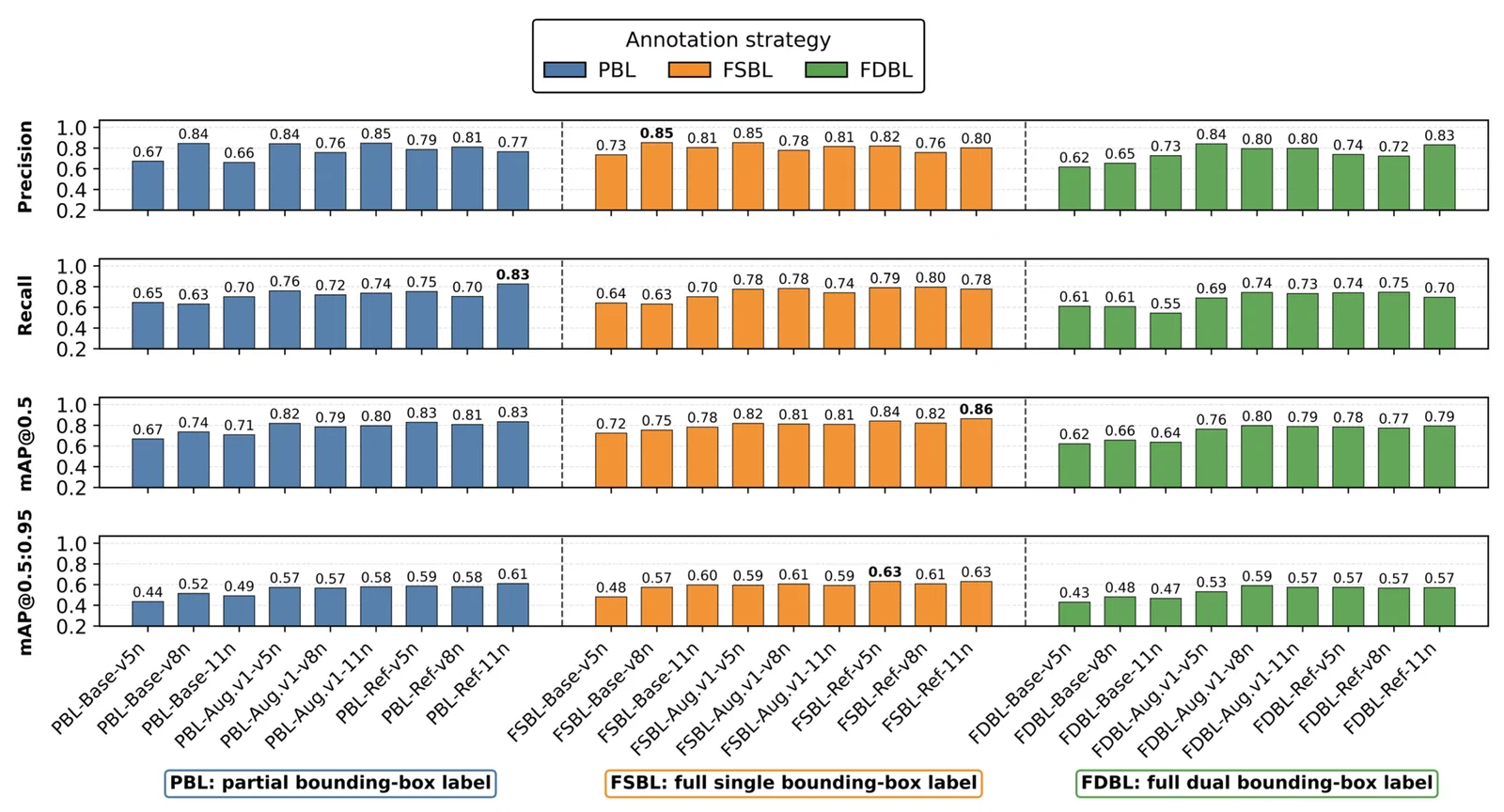
General detection metrics (precision, recall, mAP50, mAP50–95) across the 27-run matrix for PBL, FSBL and FDBL. Values in bold indicate the highest value of each metric across the 27 configurations. mAP50, mean average precision at an intersection over union threshold of 0.50; mAP50–95, mean average precision averaged over intersection over union thresholds from 0.50 to 0.95.

General detection metrics across all 27 configurations are shown in Figure 6 and reported in full in Table 6. Table 6 is evaluated on the validation split (128 images), used for checkpoint selection during training; the corresponding final performance of the selected headline configurations on the untouched test split is reported in Section 3.13 and Supplementary Table S4. The highest mAP50 in the matrix was 0.864, obtained by FSBL with YOLO11n under Refined composition across three classes. The highest precision, 0.853, was obtained by FSBL under both the YOLOv8n Baseline and YOLOv5n Augmentation v1 configurations, and the highest overall recall, 0.826, was obtained by PBL with YOLO11n under Refined composition.

Performance improved with augmentation in every annotation strategy. Under PBL, mAP50 rose from a Baseline range of 0.668 to 0.736 to a Refined composition range of 0.808 to 0.835. Under FSBL, the corresponding ranges were 0.724 to 0.783 and 0.822 to 0.864, and under FDBL, 0.622 to 0.657 and 0.773 to 0.793 across four classes. FDBL values are averaged over an expanded label space and are therefore not directly comparable with the three-class strategies.

Standing and lying were detected consistently across all configurations, with performance for these classes remaining stable irrespective of annotation strategy. Variation in overall performance was therefore driven predominantly by the estrus-interaction class.

### 3.3. Rare-Class Detection Performance

**Figure 7:**
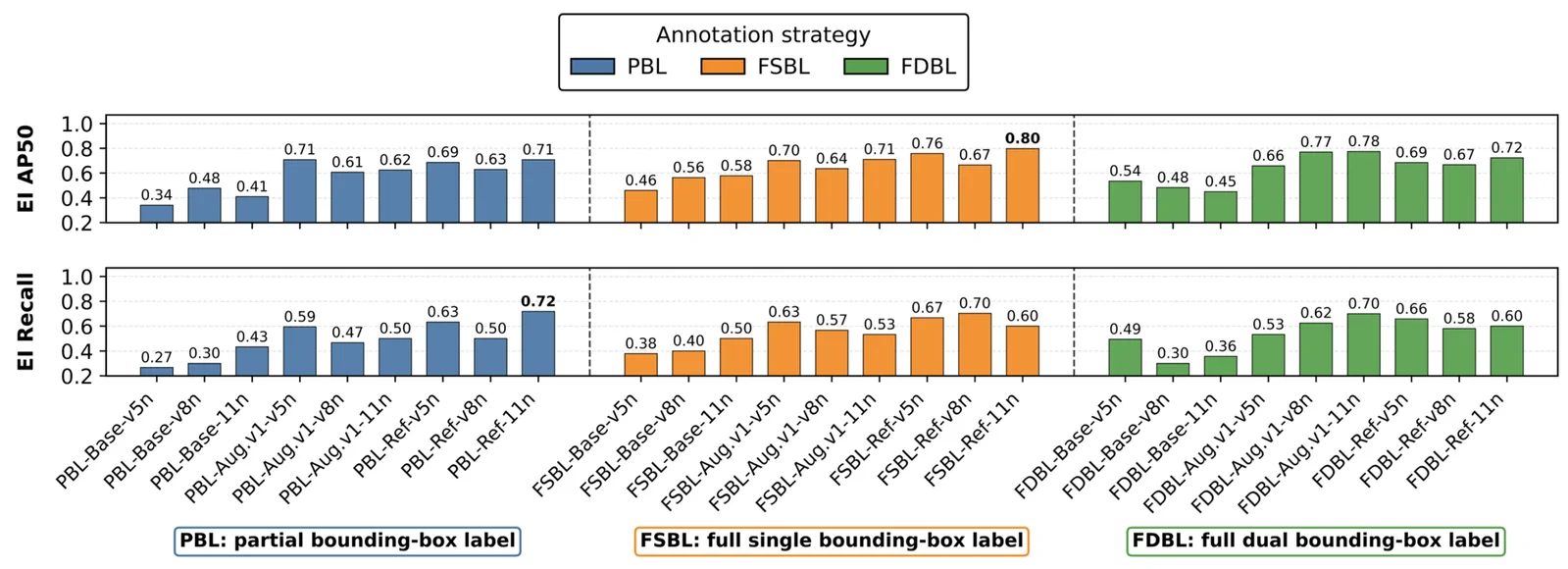
Rare-class estrus-interaction performance (EI Recall and EI AP50) across the 27-run matrix for PBL, FSBL and FDBL. Values in bold indicate the highest value of each metric across the 27 configurations.

EI Recall and EI AP50 for all 27 configurations are shown in Figure 7. The highest EI Recall was 0.718, obtained by PBL with YOLO11n under Refined composition, with a corresponding EI AP50 of 0.708. The highest EI AP50 was 0.798, obtained by FSBL with YOLO11n under Refined composition, with a corresponding EI Recall of 0.600.

Rare-class performance varied far more widely than overall performance. EI Recall ranged from 0.267 to 0.718, a spread of 0.451, and EI AP50 from 0.341 to 0.798, a spread of 0.457. Overall recall ranged from 0.545 to 0.826, a spread of 0.281, and overall mAP50 from 0.622 to 0.864, a spread of 0.242. Both rare-class metrics therefore varied approximately twice as widely across the matrix as overall mAP50. The lowest values in both rare-class metrics occurred under Baseline training, and the highest occurred under Refined composition for FSBL and for PBL EI Recall, as well as under Augmentation v1 for FDBL and for PBL EI AP50 (0.709, compared with 0.708 under Refined composition).

### 3.4. Effect of Annotation Strategy

The three strategies produced distinct performance profiles. PBL achieved the highest EI Recall in the matrix but not the highest EI AP50. FSBL achieved the highest EI AP50 and the highest overall mAP50, with an EI Recall of 0.600 in that configuration. FDBL did not exceed either strategy on any rare-class metric: its best EI Recall was 0.700 and its best EI AP50 was 0.775, both obtained with YOLO11n under Augmentation v1.

Averaged across detector families and augmentation stages, mean EI Recall was 0.490 for PBL, 0.553 for FSBL and 0.538 for FDBL, and mean EI AP50 was 0.577, 0.652 and 0.639, respectively. The strategy achieving the highest single EI Recall value was therefore not the strategy with the highest mean rare-class performance.

### 3.5. Effect of Augmentation Stage

**Figure 8:**
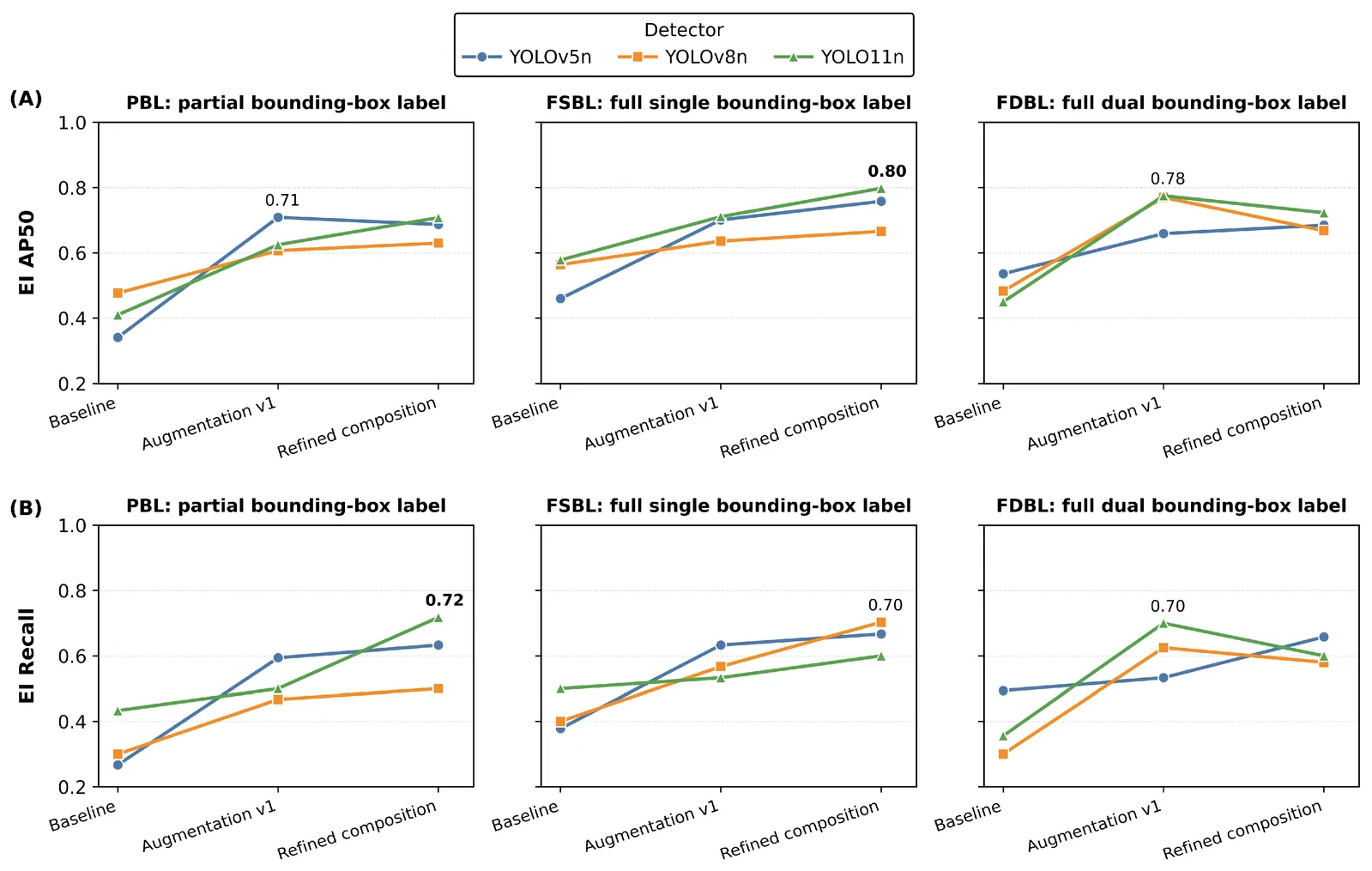
Augmentation-stage trends for the estrus-interaction class. (**A**) EI AP50; (**B**) EI Recall, for PBL, FSBL and FDBL across the three augmentation stages. Annotated values mark the highest value within each annotation strategy; values in bold indicate the highest value across all 27 configurations.

Augmentation improved rare-class performance under all three strategies, but the stage at which performance peaked differed (Figure 8). Under PBL, EI Recall rose from a Baseline range of 0.267 to 0.433, from 0.467 to 0.594 under Augmentation v1, and from 0.500 to 0.718 under Refined composition. Under FSBL, the pattern was similar, with the maximum EI AP50 of 0.798 occurring under Refined composition. The best-performing detector within each annotation strategy and augmentation stage combination is given in Table S2.

FDBL behaved differently. Its highest EI Recall of 0.700 and highest EI AP50 of 0.775 both occurred under Augmentation v1, and neither improved under Refined composition, where the corresponding maxima were 0.658 and 0.723. FDBL was therefore the only strategy for which the more elaborate augmentation policy did not produce the best rare-class result.

### 3.6. Detector Family Comparison

No detector family was uniformly superior. YOLO11n produced the highest EI Recall under PBL, the highest EI AP50 and mAP50 under FSBL, and the highest EI Recall and EI AP50 under FDBL with Augmentation v1. YOLOv5n tied with YOLOv8n for the highest precision in the matrix, both at 0.853. YOLOv8n produced the highest EI Recall under FSBL with Refined composition, at 0.703, and the highest FDBL mAP50 under Augmentation v1.

Counting the strategy and stage combinations in which each family produced the best EI Recall, YOLO11n and YOLOv5n each led in four of nine and YOLOv8n in one. The corresponding counts for EI AP50 were six, two and one. Differences between families within a given strategy and stage combination were generally smaller than differences between augmentation stages within a given family. Under PBL with YOLO11n, EI Recall increased by 0.285 from Baseline to Refined composition, whereas the largest difference between families within any single strategy and stage combination was 0.218.

### 3.7. Objective-Dependent Configuration Selection

Because no configuration maximized all metrics simultaneously, the results are summarized by monitoring objective in Table 7. The configuration achieving the highest proportion of labeled interactions detected was PBL with YOLO11n under Refined composition. The configuration achieving the most accurate rare-class localization was FSBL with YOLO11n under Refined composition. Where relational context between interacting animals was required, FDBL with YOLO11n under Augmentation v1 gave the strongest rare-class result, and FDBL with YOLOv8n under Augmentation v1 gave the highest FDBL mAP50.

### 3.8. Qualitative Observations

Inspection of prediction overlays was consistent with the quantitative results. Standing and lying were detected stably across all annotation strategies, with most errors involving marginal bounding-box placement rather than missed detections. Estrus-interaction errors were more frequent and concentrated in crowded pens, partially occluded scenes, low-contrast frames and cases in which ordinary social contact resembled estrus-associated interaction. Representative difficult cases are provided in Figure S2.

**Figure 9:**
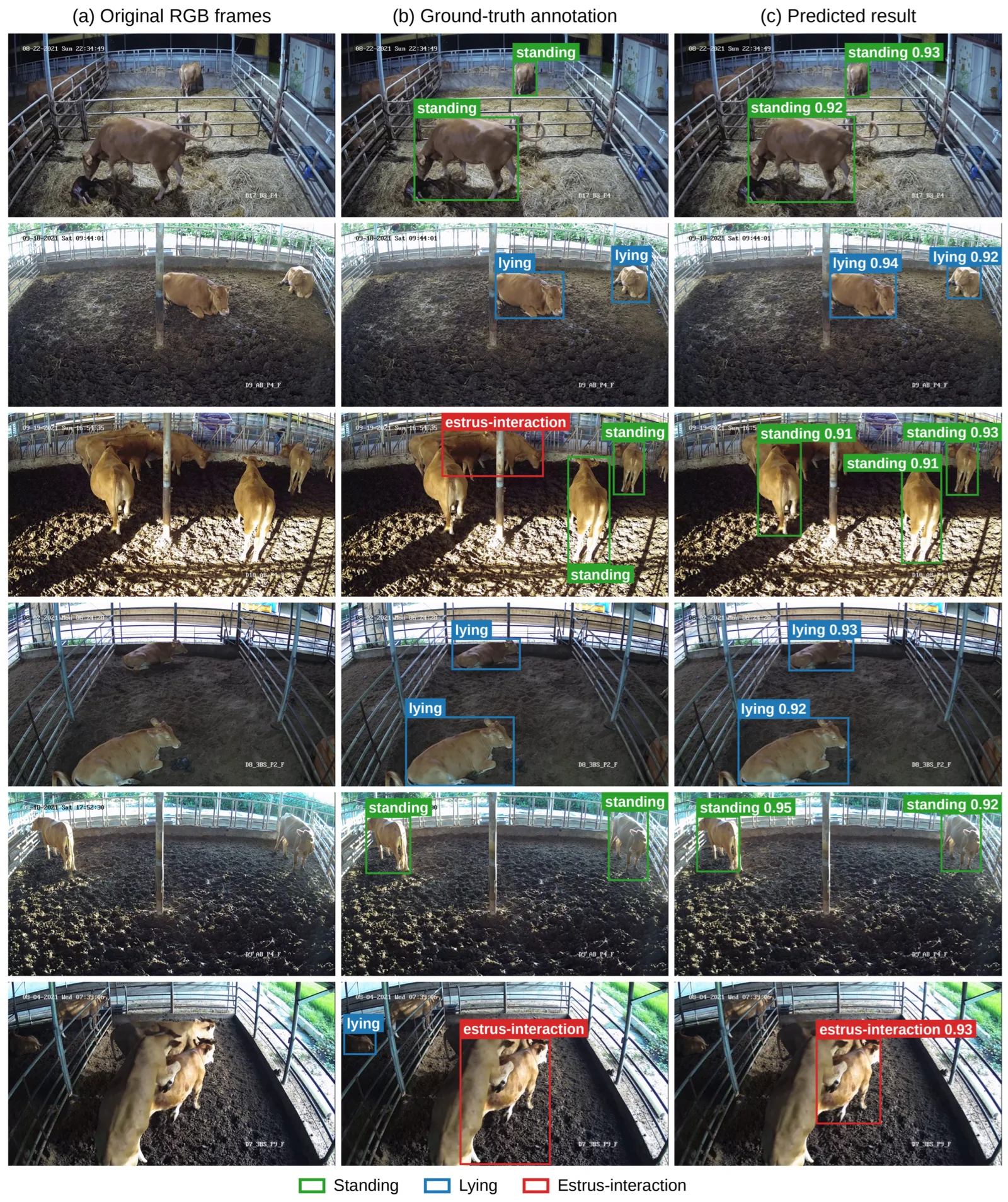
Qualitative detection pipeline for six validation frames: (**a**) original RGB frames; (**b**) ground-truth annotations; (**c**) representative predictions of PBL + YOLO11n + Refined composition at a confidence threshold of 0.25, showing class labels and confidence scores. Box colors denote the classes: green, standing; blue, lying; red, estrus-interaction. The estrus-interaction in the third row, a crowded scene, was not detected at this threshold.

The three strategies produced visibly different failure modes. PBL detections captured compact contact regions and identified some subtle cues that broader representations missed, but occasionally produced fragmented or spatially narrow boxes in crowded conditions. FSBL produced visually stable single-box predictions consistent with its stronger localization metrics. FDBL displayed both the interaction box and the companion context box, which was informative when several animals were present but harder to interpret when bodies overlapped. Representative detections are shown in Figure 9.

### 3.9. Held-Out-Enclosure Robustness Check

A census of the 197 estrus-interaction-positive frames showed that a single enclosure (designated D10_AB_P4_B) accounted for 25.4% of all estrus-interaction-positive frames in the dataset and 35.2% of the own-farm subset; the four most frequent enclosures together accounted for 77.5% of the own-farm estrus-interaction-positive subset. This enclosure’s 50 frames were distributed 38/6/6 across the training, validation and test partitions, identically for all three annotation strategies, so a portion of the reported performance could in principle reflect familiarity with this recording environment rather than the estrus-interaction cue itself.

To test this directly, the three headline configurations were retrained with the dominant enclosure’s 50 frames removed entirely from training and validation, and they were then evaluated on (a) those 50 held-out frames and (b) the remaining test frames with the enclosure’s six test frames also excluded (Table 8). A fourth configuration retrained FDBL under the Refined augmentation stage used by PBL and FSBL’s headline configurations, isolating the contribution of annotation scheme from augmentation stage.

EI Recall fell for every configuration evaluated on the held-out enclosure, most severely for PBL (−72.7% relative). Matching FDBL’s augmentation stage to Refined did not close the gap to FSBL: held-out EI Recall was slightly lower under Refined (0.256) than under FDBL’s own headline Augmentation v1 stage (0.411), indicating that augmentation-stage mismatch does not explain FDBL’s comparatively weaker held-out performance. In a representative held-out frame, the FDBL model missed the estrus-interaction box but correctly localized the companion recipient-cow box at 0.89 confidence, whereas the PBL and FSBL held-out models produced no residual detection on the same frame; this partial-detection behavior is reported as a single dataset-specific observation rather than an established property of dual-box annotation schemes.

### 3.10. Sub-Behavior Composition of Estrus-Interaction

All 200 estrus-interaction boxes in the dataset were independently reviewed and assigned to one of six candidate sub-behavior categories (standing_heat, mounting_head, mounting_rump, vulva_sniffing, chin_resting, flehmen_response), first by an automated proposal made blind to any pre-existing label and then confirmed or overridden by the first author. For the 144 boxes drawn from the 142 own-farm images, 57 boxes (39.6%) were reassigned during confirmation, yielding Cohen’s kappa = 0.384 (“fair”) and Gwet’s AC1 = 0.496 (“moderate”) agreement between the automated proposal and the confirmed label; this disagreement rate indicates that sub-behavior boundaries within the composite estrus-interaction class are genuinely ambiguous from single static frames in a meaningful fraction of cases. Review of the remaining 56 boxes additionally identified and corrected a labeling-convention inconsistency in which a small number of boxes had been tagged with a standing-posture label duplicating, rather than adding to, the mounting category used elsewhere; once corrected, both subsets use the same four-category scheme. Standing_heat and flehmen_response are inherently temporal behaviors that cannot be reliably confirmed from a single static frame under this study’s frame-level annotation protocol, and both are therefore omitted from Table 9 rather than reported as a zero-count category.

Chin-resting and mounting-rump together account for 74.5% of the reviewed instances; mounting_head (*n* = 4) is too small a sample to support independent detection at the current dataset scale. The per-sub-behavior recall of the trained detector could not be decomposed without a fully re-annotated dataset and is stated here as an explicit limitation.

### 3.11. Five-Seed Replication of Headline Configurations

The three configurations achieving the best single-seed rare-class result in the complete 27-configuration matrix (PBL + YOLO11n + Refined; FSBL + YOLO11n + Refined; FDBL + YOLO11n + Augmentation v1) were retrained with four additional random seeds, five seeds in total (seed = 0–4), and are reported as mean ± SD alongside the original seed = 0 value (Table 10; full per-seed data in Supplementary Table S3).

Five of the six originally reported seed = 0 values exceeded their five-seed mean, by 0.029–0.097 absolute; the exception was FSBL’s EI Recall, which fell 0.029 below its mean (0.600 vs. 0.629 ± 0.025). The originally reported seed = 0 values therefore did not reliably represent the center of the run-to-run distribution for these configurations. The complete 27-configuration matrix remains a single-seed comparative screen; only these three configurations were subjected to five-seed replication.

### 3.12. Effect of Mosaic and Mixup Augmentation

The Refined composition’s internal components, mosaic and mixup, were ablated across all three annotation strategies with YOLO11n, comparing the original Refined-stage setting (components on) against the same configuration with both components turned off. This ablation is reported on the validation split for direct comparability with Table 6 (Table 11); the identical eight configurations evaluated on the held-out test split are reported in Supplementary Table S4 as a robustness check, and the same qualitative pattern holds on both splits.

Disabling mosaic and mixup had a materially different, and in one case opposite-direction, effect depending on annotation strategy. PBL’s EI Recall was essentially unchanged (0.718 to 0.700), while EI AP50 improved (0.708 to 0.738); FSBL’s EI Recall was essentially unchanged (0.600 to 0.602), while EI AP50 decreased (0.798 to 0.696); FDBL showed the largest and most consistent benefit from disabling these components, on both EI Recall (0.600 to 0.783) and EI AP50 (0.723 to 0.829). The same pattern, FDBL improving substantially with mosaic and mixup off while PBL and FSBL show no comparable benefit, held in the test-split robustness check (Supplementary Table S4), despite different absolute values between splits, consistent with the checkpoint-selection validation split and the held-out test split being evaluated on different frames.

Direct verification of the training configuration files also identified that the originally reported YOLOv5n Refined-stage runs for PBL and FSBL had been trained with mixup effectively at 0.0 rather than the intended 0.1, an inconsistency disclosed here in full. Both configurations were retrained with the corrected mixup = 0.1 setting on the same framework used throughout the rest of the study, evaluated on the same validation split as the rest of this table for direct comparability: EI Recall and EI AP50 changed from 0.667/0.793 to 0.633/0.687 for PBL, and from 0.600/0.713 to 0.667/0.758 for FSBL, relative to the originally reported (flawed) values. These corrected values now also replace the two affected rows of Table 6 and are discussed further in Section 4.7.

### 3.13. Confidence-Threshold Sensitivity

A confidence-threshold sweep was run for the three headline configurations at three operating points (0.25, 0.50, 0.75) on the held-out test split (32 ground-truth EI instances in 29 images), reporting EI precision, EI Recall and EI F1 at each threshold (Table 12). These swept values apply realistic, restrictive operating thresholds and are not directly comparable in absolute terms to the near-zero-threshold headline EI Recall figures used for mAP/AP50 calculation elsewhere in this manuscript (e.g., 0.718 for PBL + YOLO11n + Refined in Table 6); the sweep characterizes practical deployment behavior, a distinct and complementary question from the comparative headline metric. For direct comparison with Table 6, using the identical near-zero-threshold AP50 convention, the three headline configurations achieved the following final performance on the untouched test split: PBL + YOLO11n + Refined, EI Recall 0.531, EI AP50 0.560, mAP50 0.763; FSBL + YOLO11n + Refined, EI Recall 0.562, EI AP50 0.682, mAP50 0.808; FDBL + YOLO11n + Augmentation v1, EI Recall 0.547, EI AP50 0.696, mAP50 0.723 (Supplementary Table S4).

All three configurations showed the classic precision/recall trade-off as the threshold was raised (e.g., FSBL precision 0.857 to 0.938 while recall fell 0.560 to 0.469). EI F1 peaked at the most permissive threshold tested (0.25) for all three configurations, indicating that, on this test partition, a lower acceptance threshold gave the best overall precision/recall balance.

## 4. Discussion

### 4.1. Annotation Strategy Determines the Sensitivity–Localization Trade-Off

The clearest finding of this evaluation is that the spatial definition of the labeled target changed which aspect of detection performance improved. PBL produced the highest proportion of labeled interactions detected, at an EI Recall of 0.718, but did not produce the highest localization quality. FSBL produced the highest EI AP50 at 0.798 and the highest overall mAP50 at 0.864, but it reached an EI Recall of only 0.600 in that configuration. The same underlying frames, the same detector and the same augmentation policy therefore yielded materially different outcomes according to how the interaction was enclosed.

The higher sensitivity observed with PBL may reflect the concentration of supervision on the contact region, where the geometry distinguishing mounting from ordinary social proximity is most apparent. A compact box excludes surrounding background from the labeled region, whereas a broader box distributes the same evidence across a larger proportion of less informative pixels. The present experiments did not isolate the contribution of this spatial mechanism, and this interpretation remains a plausible account of the observed result rather than a demonstrated cause.

The inclusive FSBL box preserves body-level structure at the cost of that concentration. Because a bounding box determines not only where the target lies but what visual evidence the detector associates with the class, a representation that consistently encloses whole participating bodies may produce more stable box regression, even if individual subtle cues become proportionally less prominent within the labeled region. Previous object-detection studies have shown that annotation quality and spatial label definition can influence detection and localization performance [22,23,24,25,33].

FDBL preserved relational context by pairing the interaction box with a box marking the recipient animal, and it did not exceed either alternative on any rare-class metric. Its best EI Recall was 0.700 and its best EI AP50 0.775, both under Augmentation v1. Richer relational annotation therefore did not improve frame-level detection under the present evaluation. The additional label increases spatial complexity precisely where the interaction is hardest to resolve, in crowded pens where the two boxes overlap, which is consistent with the qualitative observation that FDBL predictions were harder to interpret when bodies overlapped. The value of relational labeling depends on whether the downstream purpose is frame-level detection accuracy or contextual representation that could support later temporal or relational analysis [26,27].

### 4.2. Augmentation Interacts with Annotation Representation

Augmentation improved rare-class performance under all three strategies, but the stage at which performance peaked was not the same. PBL and FSBL reached their maxima under Refined composition, except for PBL EI AP50, which was effectively equal under Augmentation v1 and Refined composition (0.709 and 0.708), while FDBL reached both its highest EI Recall and its highest EI AP50 under the intermediate Augmentation v1 stage and did not improve further.

This asymmetry is informative because the two stages differ in kind, not only in degree. Augmentation v1 applied photometric and geometric variation to single images, whereas Refined composition additionally introduced mosaic and mixup at low probability, both of which combine content from multiple source images. For PBL and FSBL, which label a single spatial region, such composition appears to have increased the diversity of visual presentations available during training for a class represented by only 200 instances. For FDBL, which already encodes relational context explicitly through a second box, composite images may add spatial complexity without adding relational information that the annotation does not already carry.

The practical consequence is that augmentation composition cannot be selected independently of annotation design. This supports treating augmentation as an active experimental variable rather than a fixed preprocessing step [31,34], a distinction that the cattle-vision studies examined here do not make. Targeted augmentation composition has previously been shown to reduce estimation error substantially in domain-limited agricultural detection tasks where parameters were optimized deliberately rather than left at framework defaults [34], and the present results extend that observation to a sparsely represented interaction class.

### 4.3. Detector Family Was Not the Dominant Factor

Detector family contributed to performance differences but did not determine them. No family was uniformly superior. YOLO11n and YOLOv5n each produced the best EI Recall in four of the nine strategy and stage combinations, and YOLOv8n did so in one. For EI AP50, the counts were six, two and one, respectively. YOLOv5n, the oldest architecture evaluated, tied with YOLOv8n for the highest precision in the entire matrix, both at 0.853.

The magnitude of the effects is more informative than the counts. Moving PBL with YOLO11n from Baseline to Refined composition raised EI Recall by 0.285, whereas the largest difference between detector families within any single strategy and stage combination was 0.218. Across the matrix, EI Recall ranged from 0.267 to 0.718 and EI AP50 from 0.341 to 0.798, spreads of 0.451 and 0.457, respectively, while overall mAP50 ranged from 0.622 to 0.864, a spread of 0.242. Generational advancement in detector architecture therefore did not guarantee proportional gains under imbalanced rare-event conditions.

This does not diminish the value of architectural work. It indicates that under the controlled conditions tested, the performance differences associated with annotation and augmentation choices were larger than those associated with changing detector family, and that detector selection is best treated as one component of an integrated annotation, augmentation and model decision.

### 4.4. Comparison with Previous Cattle-Vision Studies

The present results are not directly comparable with studies reporting high accuracy for cattle behavior recognition, because the problem structures differ. Multi-view action recognition in closed barns has been performed on 112,761 annotated bounding boxes across eleven action classes [13], and lightweight detectors have recognized four posture classes from 5051 images [14]. Both address behaviors of individual animals with substantial training data per class. The present dataset contains 200 instances of the target interaction class distributed across 197 images, substantially fewer positive examples of a single behavior than are available in the multi-class cattle behavior datasets used in previous work.

The same scarcity has been encountered in video-based approaches. A beef cattle abnormal behavior dataset comprising 1285 clips contained 176 mounting clips against 931 normal-behavior clips, and class imbalance was addressed through a modified loss function rather than through data representation [35]. That study operated on clip-level labels and predicted the behavior class of an entire video segment, whereas the present work requires spatial localization within individual frames. The two approaches therefore represent complementary responses to the same underlying difficulty: a behavior of clinical interest that occurs rarely relative to routine activity.

Camera-based estrus detection has also been demonstrated previously, but under conditions that simplify the visual problem considerably. Background subtraction of whole-animal movement identified estrus-associated activity increases in tie-stalled cows using one camera view per animal and no learned detector [12]. Thermal tracking in Hanwoo detected a 2.35-fold increase in movement and an 11% reduction in feed intake on the day of estrus [8]. Both infer estrus from aggregate change in activity or intake rather than from the interactions themselves, and both avoid the occlusion and animal-assignment problems inherent to group housing. Work on Hanwoo imagery has otherwise concentrated on identity, through muzzle-pattern identification [15] and cross-view re-identification [16], which supports long-term monitoring of individual animals but addresses a different target.

The contribution of the present work is therefore not a higher accuracy figure but a characterization of how the detection problem should be formulated when the target is rare and relational. Reported accuracy on a large, balanced, single-class dataset does not indicate how a system will behave when the class of interest constitutes 8% of annotations and involves two overlapping animals.

### 4.5. Implications for Camera-Based Estrus Monitoring

The finding that no configuration maximized every metric has a direct consequence for system design: the appropriate configuration depends on what the farm intends to do with the output.

Where the objective is screening, and the cost of missing a candidate interaction exceeds the cost of reviewing a false positive, PBL with YOLO11n under Refined composition detected the highest proportion of labeled interactions at an EI Recall of 0.718. Candidate detections from such a system are reviewed or filtered downstream, so the system could support rather than replace observational labor. This distinction is particularly relevant because standing to be mounted is not expressed in every estrous event, which increases the potential value of complementary interaction cues for candidate-event screening. Where the objective is confirmation support, and detections must be spatially accurate enough for an operator or veterinarian to interpret, FSBL with YOLO11n under Refined composition produced the most accurate localization at an EI AP50 of 0.798. Where relational context between interacting animals is required for subsequent behavioral analysis, FDBL under Augmentation v1 preserves that context at competitive rare-class performance.

Although the dataset comprises Hanwoo cows, the design questions addressed here are not primarily breed-specific. Coat pattern and texture strongly influence tasks that depend on surface appearance, and Hanwoo cows have been described as weakly textured with limited discriminative cues for individual identification [15,16]. The present target is different: mounting, chin-resting and sniffing are recognized from the spatial relationship between two bodies rather than from coat appearance. The trade-off between sensitivity and localization, the ambiguity of the target’s spatial extent, and the scarcity of positive examples therefore arise wherever such interactions are detected from group-housed cattle imagery, and the annotation and augmentation findings should transfer to other breeds recorded under comparable conditions, although absolute performance may differ with coat contrast against the pen background.

Detection metrics alone are an incomplete basis for deployment decisions. It has been recommended that reported performance be accompanied by threshold-dependent operational summaries, such as expected alerts per day or false positives per 100 cow-days at a stated operating threshold [11]. The present study evaluated individual frames rather than continuous monitoring, and daily alert burden cannot be estimated from these data. Aggregating frame-level detections into event-level alerts, and quantifying the resulting review workload under practical stocking and lighting conditions, is a necessary step before farm deployment.

The relationship between what the system detects and what a farm needs to know also requires care. Estrus is a physiological state expressed over several hours, whereas this system detects individual frames containing interaction cues. Frame-level detection is best understood as the sensing layer of a monitoring pipeline, within which temporal aggregation and, depending on the intended application, animal identification or physiological confirmation could be incorporated before a reproductive decision is taken. Recent assessments of precision livestock farming have argued that the field’s principal need is no longer new methods but validated guidance that can be implemented on commercial farms [10,36], and cost, data interoperability and generalizability across environments have been identified as the principal barriers to adoption [37]. The objective-dependent recommendations presented here are intended to serve that purpose for camera-based interaction detection.

### 4.6. Robustness to Unseen Enclosures and Sub-Behavior Composition

The held-out-enclosure check indicates that a meaningful share of the headline rare-class performance reflects familiarity with recording-specific visual conditions rather than the estrus-interaction cue alone, most severely for PBL. A comparable magnitude of location-held-out degradation has been reported elsewhere in ecological computer vision, where camera-trap species-classification accuracy has been reported to fall substantially when evaluated on recording locations absent from training [38], although no direct numerical comparison across studies, tasks and evaluation protocols is implied here. Annotation-box area provides a plausible, though not causally isolated, contributing mechanism: PBL’s estrus-interaction boxes average 10.1% of frame area across the dataset (12.6% within the held-out enclosure) versus 13.6% for FSBL and FDBL (17.7% within the held-out enclosure), consistent with evidence that object detectors learn foreground–background context associations during training that can fail to transfer under distribution shift [39,40]. Because FDBL’s estrus-interaction box is identical in size to FSBL’s, FDBL’s comparatively weaker held-out generalization reflects a property of the dual-box annotation scheme itself rather than box geometry. Annotation-box strategy should therefore be selected considering not only headline within-distribution accuracy but also robustness to unseen enclosures: where cross-farm robustness is the primary objective, FSBL is the strongest recommendation from this experiment, while FDBL may still offer practical value through its companion-class partial signal under distribution shift despite weaker primary-class recall under the same conditions.

Chin-resting and anogenital sniffing (vulva_sniffing) are independently established secondary estrus signs that, together with mounting, form a classic secondary-sign triad in the cattle ethology literature [41]. Mounting orientation has also been treated as a distinguishable behavioral variable in cattle studies, including head-side or head mounting in Holstein cattle [42] and disoriented mounting in Carora dairy cows [43]. Thus, distinguishing mounting orientation in the present post hoc classification is supported by previously documented cattle estrus behaviors rather than representing an ad hoc classification specific to this study.

### 4.7. Seed Sensitivity and the Corrected YOLOv5n Refined-Stage Runs

The five-seed replication indicates that single-seed comparisons among closely matched configurations should be interpreted cautiously. With a test set of 32 estrus-interaction instances, a difference of two to three correctly detected boxes shifts EI Recall by approximately 6–9 percentage points; this discrete-count sensitivity is specific to recall and does not automatically transfer to the same magnitude for AP50, which aggregates precision across recall levels. Seed-driven score differences of comparable magnitude have been shown elsewhere to be sufficient to change which of two systems appears state-of-the-art [44], reinforcing current reporting standards that treat uncertainty quantification as an explicit expectation rather than an optional addition.

During preparation of this revision, direct verification of the training configuration files identified that the originally reported YOLOv5n Refined-stage runs for PBL and FSBL had been trained with mixup effectively at 0.0 rather than the intended 0.1, while the corresponding FDBL run used mixup at 0.1 on a different underlying training framework. Both configurations were retrained with the corrected mixup = 0.1 setting on the framework used throughout the rest of the study and evaluated on the validation split used for checkpoint selection throughout this manuscript, for direct comparability with the original values: corrected PBL + YOLOv5n + Refined now yields EI Recall 0.633 and EI AP50 0.687 (overall mAP50 0.829), compared with the originally reported 0.667 and 0.793 (0.855); corrected FSBL + YOLOv5n + Refined yields EI Recall 0.667 and EI AP50 0.758 (overall mAP50 0.841), compared with the originally reported 0.600 and 0.713 (0.831). These corrected values have replaced the two affected rows of Table 6 and are also reported alongside the mosaic/mixup ablation in Table 11 for direct comparison on the same split.

### 4.8. Relational Annotation, Deployment Threshold and Camera Viewpoint

At the standard YOLO detection head, FDBL outputs two independent, co-occurring bounding-box predictions, the estrus-interaction box and the companion recipient-cow box, with no built-in associative mechanism linking a specific interaction box to a specific recipient box when multiple animals are in close proximity; FDBL does not construct an integrated relational graph. This is consistent with the design of recently published cattle- and livestock-interaction computer-vision pipelines, which detect individual animals first and derive pairwise interactions as an explicit secondary step: a triple-stream cattle-interaction network constructs interaction proposals from pairs of individually detected animal boxes using an intersection-over-union criterion [45], a recent cattle-interaction system tracks individually detected animals before forming interaction candidates from overlapping tracked boxes [46], and a black-beef-cattle mounting-detection system derives interacting-pair regions from the intersection of independently detected single-animal regions [21]. Realizing FDBL’s full practical value for behavior-relationship modeling or individual-animal tracking would require adding an explicit secondary association step, such as spatial affinity, a tracking identifier, or a lightweight graph-based pairing model, which is proposed here as a specific direction for future work. FDBL’s estrus-interaction box is identical in size to FSBL’s, so box geometry does not explain its weaker frame-level performance; the residual gap is attributed to FDBL’s more difficult joint learning problem, in which the detector must learn two independently supervised classes per positive instance on an already rare-event dataset. System designers should select FSBL when frame-level detection accuracy for the interaction event itself is the primary objective, and they should consider FDBL, paired with an explicit association step, when the downstream system additionally requires individual identity, tracking or relational reasoning about which specific animals were involved.

Because EI F1 peaked at the most permissive threshold tested (Section 3.13) and a missed genuine estrus-interaction event carries a higher practical cost than an extra event flagged for operator review, a confidence threshold near 0.25 is recommended as the default operating point for on-farm deployment, with 0.50 offered as a higher-precision alternative where reducing operator alert volume is prioritized over maximizing sensitivity. Operators should treat the confidence threshold as a deployment-time tuning parameter rather than a fixed default.

Qualitative inspection additionally suggested that camera viewpoint and stocking density contributed to false-positive estrus-interaction detections: close, sustained social contact between similarly posed animals viewed from an oblique or partially occluded angle was, in a subset of cases, visually difficult to distinguish from mounting or chin-resting, particularly in more densely stocked pens. This observation is consistent with reports that camera placement and barn layout are determinants of estrus-detection performance [11] and suggests that camera positioning that reduces oblique viewing angles on high-density pens is a practical, low-cost step toward reducing this class of false positive in deployment.

### 4.9. Limitations

The dataset was recorded at a single commercial farm, and the results do not constitute cross-farm validation. Recording nonetheless spanned multiple pens and seasons, so the footage included substantial variation in illumination, animal density, pen structure, flooring and animal distance from the camera, including infrared night-time recording. Differences in barn design, camera position and management practice may still alter performance elsewhere. The partition was applied at frame level rather than by recording session or pen, so the comparison across configurations is controlled but the test subset was not drawn from wholly independent recordings; partitioning by pen or session would provide a stricter test of generalization. Limited generalizability of trained models across farm environments is a recognized constraint in precision livestock farming rather than one specific to this dataset [9,29,47], and camera placement and barn layout have been identified as determinants of estrus-detection performance [11]. Evaluation across multiple farms, as well as across breeds differing in coat appearance, is the most important next step.

The estrus-interaction class combined mounting, chin-resting and sniffing into a single operational category. These cues are visually distinguishable but were grouped because they occur within the same behavioral context and are individually too infrequent for separate modeling at this dataset scale. The design therefore evaluates detection of a composite behavioral proxy rather than the independent diagnostic value of each cue, and the relative contribution of the three components to overall class performance was not assessed.

The study was confined to frame-level detection. No sequence modeling, temporal event linking or physiological confirmation was performed, and detections should be read as visual interaction cues rather than confirmed reproductive events. The rare class remained small relative to the posture classes, and performance may be sensitive to additional annotated examples, to annotation consistency and to greater scene diversity.

Because a fixed data partition and a single training run were used for 24 of the 27 configurations, the reported differences for those configurations describe comparative performance under controlled conditions rather than statistically estimated differences; variability between repeated training runs was quantified only for the three configurations subjected to five-seed replication (Section 3.11). Differences of the magnitude observed between detector families, at most 0.218 in EI Recall within a strategy and stage combination, should therefore be interpreted with more caution than the larger differences observed between augmentation stages. The conclusions describe the relative behavior of these design choices under the present experimental conditions rather than a universal ranking.

The held-out-enclosure check (Section 3.9) removed only a single dominant enclosure; robustness to a wider range of unseen recording environments remains to be established, and the FDBL companion-box observation reported there has not been shown to support recovery of a missed interaction, only partial residual signal, in a single representative frame.

The sub-behavior composition reported in Section 3.10 is a static, box-level classification of the existing annotations and does not decompose the trained detector’s own per-sub-behavior recall; a fully re-annotated dataset would be required to do so.

## 5. Conclusions

This study evaluated 27 configurations combining three annotation strategies, three augmentation stages and three lightweight detector families for frame-level detection of estrus-related interaction behavior in group-housed Hanwoo cattle, using a fixed dataset partition and identical training conditions throughout. Detection of the rare interaction class varied far more widely across the matrix than detection of the frequent posture classes, and the source of that variation was predominantly the formulation of the detection problem rather than the choice of detector. Compact interaction-focused labeling detected the highest proportion of labeled interactions, at an estrus-interaction recall of 0.718, whereas inclusive single-box labeling produced the most accurate localization, at an estrus-interaction AP50 of 0.798 and an overall mAP50 of 0.864. Dual-box labeling preserved relational context between interacting animals at competitive performance but did not exceed either alternative on any rare-class metric. Augmentation improved rare-class detection under all three strategies, but the stage at which performance peaked depended on the annotation representation used.

These findings indicate that annotation design and augmentation composition should be treated as experimental variables selected according to the intended monitoring objective, rather than as fixed preprocessing decisions made before model selection. For screening, where the aim is to capture as many candidate interactions as possible for subsequent review, sensitivity-oriented labeling is appropriate; for confirmation support, where detections must be spatially accurate enough for expert interpretation, localization-oriented labeling is preferable. Detections represent a behavioral proxy for estrus-related interaction and do not constitute physiological confirmation of estrus. Evaluation across multiple farms, aggregation of frame-level detections into event-level alerts, and integration with complementary sensing would be required before deployment in commercial reproductive management.

## Figures and Tables

**Table 1:** Composition of the locked Hanwoo dataset.

Subset	Total Images	Labeled Images	Background-Only Images	Purpose
Training	593	552	41	Model training
Validation	128	119	9	Early stopping and metric tracking
Test	125	117	8	Held-out final reporting
Total	846	788	58	197 images contain estrus-interaction

Of the 197 images containing at least one estrus-interaction instance, 138 fall in the training subset, 30 in validation and 29 in test. Section 3.2 reports the main 27-configuration comparison on the validation split, used for checkpoint selection during training; the test split is held out throughout training and selection, and is used for final performance reporting in Section 3.13 and Supplementary Table S4.

**Table 2:** Definition and intended role of the three annotation strategies.

Strategy	Representation	Box Composition	Intended Role
PBL	Partial, event-focused	Tight box on the interaction zone	Minimize background burden; isolate rare-event signal
FSBL	Full single bounding-box	One inclusive box covering the interaction region	Provide explicit behavioral labeling without dual representation
FDBL	Full dual bounding-box	Estrus-interaction box plus recipient-cow box	Encode both the event and surrounding behavioral context

PBL, partial bounding-box label; FSBL, full single bounding-box label; FDBL, full dual bounding-box label.

**Table 3:** Class-instance counts under each annotation strategy.

Strategy	Standing	Lying	Estrus-Interaction	Recipient Cow	Total
PBL	1590	590	200	–	2380
FSBL	1590	590	200	–	2380
FDBL	1590	590	200	200	2580

PBL, partial bounding-box label; FSBL, full single bounding-box label; FDBL, full dual bounding-box label.

**Table 4:** Training configuration applied across all 27 runs.

Category	Item	Setting
Models	Detector families	YOLOv5n, YOLOv8n, YOLO11n
Input	Image size	640 × 640
Training	Epochs	150
Training	Early-stopping patience	30
Training	Batch size	8
Training	Random seed	0
Training	Deterministic mode	Enabled
Split control	Dataset partition	593/128/125 (Train/Validation/Test)
Optimization	Optimizer and schedule	Framework default per family
Inference	Non-maximum suppression intersection over union (IoU)	0.7
Hardware	Workstation	Intel Core i9-10900KF; 64 GB RAM; NVIDIA RTX 2070, 8 GB
Software	Python	3.11.1
Software	PyTorch	2.7.1
Software	CUDA	11.8
Software	Ultralytics	8.4.30

**Table 5:** Augmentation-stage design: policy, aim and rationale.

Stage	Policy	Aim	Rationale
Baseline	No augmentation	Establish reference behavior	Clean detector baseline without synthetic variability
Augmentation v1	Moderate single-image transforms	Improve robustness	Test restrained augmentation under practical farm-scene variation
Refined composition	Targeted compositional enrichment	Improve rare-class learnability	Strengthen estrus-interaction diversity while limiting unrealistic distortion

**Table 6:** Detection performance across all 27 configurations.

Strategy	Detector	Stage	Precision	Recall	mAP50	mAP50–95	EI Recall	EI AP50
PBL	YOLOv5n	Baseline	0.673	0.648	0.668	0.436	0.267	0.341
PBL	YOLOv8n	Baseline	0.845	0.633	0.736	0.515	0.300	0.477
PBL	YOLO11n	Baseline	0.662	0.703	0.709	0.492	0.433	0.410
PBL	YOLOv5n	Augmentation v1	0.842	0.760	0.819	0.573	0.594	0.709
PBL	YOLOv8n	Augmentation v1	0.757	0.722	0.785	0.566	0.467	0.607
PBL	YOLO11n	Augmentation v1	0.848	0.738	0.796	0.578	0.500	0.625
PBL	YOLOv5n	Refined composition	0.787	0.754	0.829	0.586	0.633	0.687
PBL	YOLOv8n	Refined composition	0.811	0.705	0.808	0.580	0.500	0.630
PBL	YOLO11n	Refined composition	0.765	0.826	0.835	0.611	0.718	0.708
FSBL	YOLOv5n	Baseline	0.735	0.643	0.724	0.481	0.378	0.460
FSBL	YOLOv8n	Baseline	0.853	0.633	0.754	0.574	0.400	0.564
FSBL	YOLO11n	Baseline	0.805	0.703	0.783	0.597	0.500	0.578
FSBL	YOLOv5n	Augmentation v1	0.853	0.776	0.819	0.595	0.633	0.701
FSBL	YOLOv8n	Augmentation v1	0.779	0.783	0.812	0.606	0.567	0.636
FSBL	YOLO11n	Augmentation v1	0.815	0.741	0.810	0.592	0.533	0.711
FSBL	YOLOv5n	Refined composition	0.819	0.790	0.841	0.632	0.667	0.758
FSBL	YOLOv8n	Refined composition	0.757	0.796	0.822	0.608	0.703	0.666
FSBL	YOLO11n	Refined composition	0.802	0.777	0.864	0.630	0.600	0.798
FDBL	YOLOv5n	Baseline	0.617	0.611	0.622	0.431	0.494	0.536
FDBL	YOLOv8n	Baseline	0.654	0.607	0.657	0.481	0.300	0.483
FDBL	YOLO11n	Baseline	0.727	0.545	0.637	0.467	0.356	0.450
FDBL	YOLOv5n	Augmentation v1	0.841	0.691	0.763	0.532	0.533	0.659
FDBL	YOLOv8n	Augmentation v1	0.795	0.744	0.797	0.590	0.625	0.770
FDBL	YOLO11n	Augmentation v1	0.797	0.734	0.788	0.574	0.700	0.775
FDBL	YOLOv5n	Refined composition	0.738	0.742	0.784	0.574	0.658	0.685
FDBL	YOLOv8n	Refined composition	0.723	0.747	0.773	0.567	0.580	0.668
FDBL	YOLO11n	Refined composition	0.831	0.698	0.793	0.571	0.600	0.723

PBL, partial bounding-box label; FSBL, full single bounding-box label; FDBL, full dual bounding-box label; EI, estrus-interaction; AP50, average precision at an intersection over union threshold of 0.50; mAP, mean average precision. FDBL values are averaged over four classes; PBL and FSBL over three. Because FDBL evaluates mAP over an expanded four-class label space while PBL and FSBL use three, raw mAP values are not directly comparable across annotation strategies; comparative claims across strategies rely on EI AP50 and EI Recall, which are computed per class regardless of the surrounding class set.

**Table 7:** Recommended configuration by monitoring objective.

Objective	Recommended Configuration	EI Recall	EI AP50	mAP50	Basis
Maximize rare-event sensitivity	PBL + YOLO11n + Refined composition	0.718	0.708	0.835	Highest EI Recall in the matrix
Maximize localization quality and overall balance	FSBL + YOLO11n + Refined composition	0.600	0.798	0.864	Highest EI AP50 and highest mAP50
Maximize relational rare-class performance	FDBL + YOLO11n + Augmentation v1	0.700	0.775	0.788	Strongest FDBL rare-class result
Balanced relational configuration	FDBL + YOLOv8n + Augmentation v1	0.625	0.770	0.797	Highest FDBL mAP50

PBL, partial bounding-box label; FSBL, full single bounding-box label; FDBL, full dual bounding-box label; EI, estrus-interaction. As in Table 6, FDBL’s mAP50 is averaged over four classes and is not directly comparable to the three-class mAP50 reported for PBL and FSBL; the recommendations above rely on EI Recall and EI AP50 for cross-strategy comparison.

**Table 8:** Held-out-enclosure robustness check for the three headline configurations and one deconfounding run.

Configuration	Locked EI Recall/AP50	Held-out-Enclosure EI Recall/AP50 (Δ Recall)	General, Enclosure-Excluded EI Recall/AP50
PBL + YOLO11n + Refined	0.718/0.708	0.196/0.428 (−72.7%)	0.480/0.387
FSBL + YOLO11n + Refined	0.600/0.798	0.484/0.645 (−19.4%)	0.670/0.667
FDBL + YOLO11n + Aug. v1 (headline)	0.700/0.775	0.411/0.672 (−41.3%)	0.640/0.601
FDBL + YOLO11n + Refined (deconfounding run)	0.469/0.589	0.256/0.549 (−45.3%)	0.496/0.552

PBL, partial bounding-box label; FSBL, full single bounding-box label; FDBL, full dual bounding-box label; EI, estrus-interaction; Aug. v1, Augmentation v1; Δ Recall, relative change in EI Recall from the locked value. The held-out enclosure contributed approximately 51 ground-truth EI instances.

**Table 9:** Confirmed sub-behavior composition of the 200 estrus-interaction instances.

Sub-Behavior	Confirmed Count (of 200)	Share
chin_resting	85	42.5%
mounting_rump	64	32.0%
vulva_sniffing (anogenital sniffing)	47	23.5%
mounting_head	4	2.0%

**Table 10:** Five-seed mean ± standard deviation (SD) for the three best-performing configurations, compared with the originally reported seed = 0 value.

Configuration	EI AP50 Mean ± SD	EI AP50 (Seed = 0)	EI Recall Mean ± SD	EI Recall (Seed = 0)
PBL + YOLO11n + Refined	0.679 ± 0.049	0.708	0.621 ± 0.079	0.718
FSBL + YOLO11n + Refined	0.728 ± 0.055	0.798	0.629 ± 0.025	0.600
FDBL + YOLO11n + Aug. v1	0.732 ± 0.056	0.775	0.669 ± 0.050	0.700

**Table 11:** Effect of disabling mosaic and mixup augmentation, by annotation strategy (YOLO11n, validation split), together with the corrected YOLOv5n Refined-stage mixup values (Section 4.7).

Strategy	Mosaic/Mixup	EI Recall	EI AP50	Overall mAP50
PBL	On (Refined)	0.718	0.708	0.835
PBL	Off	0.700	0.738	0.855
FSBL	On (Refined)	0.600	0.798	0.864
FSBL	Off	0.602	0.696	0.834
FDBL	On (Refined)	0.600	0.723	0.793
FDBL	Off	0.783	0.829	0.821
PBL, YOLOv5n Refined (mixup corrected)	On (Refined)	0.633	0.687	0.829
FSBL, YOLOv5n Refined (mixup corrected)	On (Refined)	0.667	0.758	0.841

PBL, partial bounding-box label; FSBL, full single bounding-box label; FDBL, full dual bounding-box label; EI, estrus-interaction. FDBL’s mAP50 is averaged over four classes and is not directly comparable to the three-class mAP50 reported for PBL and FSBL. The final two rows are the corrected YOLOv5n Refined-stage values from Section 4.7, included here for direct comparison on the same validation split; they are not part of the on/off mosaic/mixup ablation itself.

**Table 12:** Confidence-threshold sweep for the three headline configurations (held-out test split, 32 EI instances).

Configuration	Confidence Threshold	EI Precision	EI Recall	EI F1
PBL + YOLO11n + Refined	0.25	0.671	0.531	0.593
PBL + YOLO11n + Refined	0.50	0.696	0.500	0.582
PBL + YOLO11n + Refined	0.75	0.778	0.438	0.560
FSBL + YOLO11n + Refined	0.25	0.857	0.560	0.678
FSBL + YOLO11n + Refined	0.50	0.850	0.531	0.654
FSBL + YOLO11n + Refined	0.75	0.938	0.469	0.625
FDBL + YOLO11n + Aug. v1	0.25	0.818	0.562	0.667
FDBL + YOLO11n + Aug. v1	0.50	0.850	0.531	0.654
FDBL + YOLO11n + Aug. v1	0.75	0.875	0.438	0.583

## Data Availability

The data presented in this study are available on request from the corresponding author. The privately collected farm-surveillance images are not publicly available because they were recorded under institutional and laboratory data-use restrictions. The public frames used to supplement the dataset were obtained from the Open AI Dataset Project (AI-Hub, Republic of Korea) and are available at https://www.aihub.or.kr (accessed on 23 August 2026) subject to that platform’s access conditions and licensing terms. Derived experimental summaries, model-performance tables and custom scripts for annotation regeneration and metric extraction are available from the corresponding author on reasonable request.

## Supplementary Materials

The following supporting information can be downloaded at: https://www.mdpi.com/article/10.3390/ani16193054/s1, Table S1: Locked augmentation parameter values applied at each stage; Table S2: Best-performing detector within each annotation strategy and augmentation stage; Table S3: Per-seed estrus-interaction (EI) AP50 and EI Recall for the five-seed replication of the three headline configurations (PBL + YOLO11n + Refined, FSBL + YOLO11n + Refined, FDBL + YOLO11n + Augmentation v1), seeds 0–4 (Section 3.11); Table S4: Effect of disabling mosaic and mixup augmentation, by annotation strategy (YOLO11n, held-out test split), together with the corrected YOLOv5n Refined-stage mixup values evaluated on the same test split, reported as a robustness check against Table 11 (main text, validation split) (Section 3.12); Figure S1: Representative frames showing chin-resting or sniffing occurring near mounting within the same camera scene; Figure S2: Representative difficult cases for estrus-interaction detection, including crowding, partial occlusion, overlapping animals, low light and ambiguous social contact.

## Abbreviations

The following abbreviations are used in this manuscript:

AP	Average precision
EI	Estrus-interaction
FDBL	Full dual bounding-box label
FN	False negative
FP	False positive
FSBL	Full single bounding-box label
IoU	Intersection over union
mAP	Mean average precision
PBL	Partial bounding-box label
RGB	Red, green, blue
TP	True positive
YOLO	You only look once
