# Supplementary material for: Vision-Based Detection of Estrus-Related Interaction Behavior in Hanwoo Cattle Using Lightweight YOLO Models
Source: Animals (Basel). 2026 Sep 28;16(19):3054. doi: 10.3390/ani16193054 (PMC13663419; doi:10.3390/ani16193054)
Supplement: Supplementary file 1 [file animals-16-03054-s001.zip › animals-4551713-supplementary.pdf]

Supplementary Materials

Vision-Based Detection of Estrus-Related Interaction Behavior in Hanwoo Cattle Using Lightweight YOLO Models

Table S1. Locked augmentation parameter values applied at each stage.

Parameter	Baseline	Augmentation v1	Refined composition
hsv_h	0.0	0.015	0.015
hsv_s	0.0	0.35	0.32
hsv_v	0.0	0.20	0.18
degrees	0.0	0.0	0.0
translate	0.0	0.08	0.08
scale	0.0	0.20	0.18
shear	0.0	0.0	0.0
perspective	0.0	0.0	0.0
flipud	0.0	0.0	0.0
fliplr	0.0	0.5	0.5
mosaic	0.0	0.0	0.2
mixup	0.0	0.0	0.1
copy_paste	0.0	0.0	0.0
erasing	0.0	0.0	0.0

Values shown are those applied in the Ultralytics implementation (YOLOv8n, YOLO11n). Mosaic was closed during the final ten epochs of training at every stage.

Table S2. Best-performing detector within each annotation strategy and augmentation stage.

Stage	Strategy	Best EI Recall	Best EI AP50	Best mAP50
Baseline	PBL	YOLO11n, 0.433	YOLOv8n, 0.477	YOLOv8n, 0.736
Baseline	FSBL	YOLO11n, 0.500	YOLO11n, 0.578	YOLO11n, 0.783
Baseline	FDBL	YOLOv5n, 0.494	YOLOv5n, 0.536	YOLOv8n, 0.657
Augmentation v1	PBL	YOLOv5n, 0.594	YOLOv5n, 0.709	YOLOv5n, 0.819
Augmentation v1	FSBL	YOLOv5n, 0.633	YOLO11n, 0.711	YOLOv5n, 0.819
Augmentation v1	FDBL	YOLO11n, 0.700	YOLO11n, 0.775	YOLOv8n, 0.797
Refined composition	PBL	YOLO11n, 0.718	YOLO11n, 0.708	YOLO11n, 0.835
Refined composition	FSBL	YOLOv8n, 0.703	YOLO11n, 0.798	YOLO11n, 0.864
Refined composition	FDBL	YOLOv5n, 0.658	YOLO11n, 0.723	YOLO11n, 0.793

PBL, partial bounding-box label; FSBL, full single bounding-box label; FDBL, full dual bounding-box label; EI, estrus-interaction.

Table S3. Per-seed estrus-interaction (EI) AP50 and EI Recall for the five-seed replication of the three headline configurations (PBL + YOLO11n + Refined, FSBL + YOLO11n + Refined, FDBL + YOLO11n + Augmentation v1), seeds 0–4.

Configuration	Seed	EI AP50	EI Recall
PBL	0	0.706	0.703
PBL	1	0.676	0.608
PBL	2	0.639	0.495
PBL	3	0.627	0.633

PBL	4	0.746	0.667
FSBL	0	0.821	0.626
FSBL	1	0.702	0.619
FSBL	2	0.693	0.667
FSBL	3	0.689	0.600
FSBL	4	0.733	0.633
FDBL	0	0.776	0.700
FDBL	1	0.747	0.658
FDBL	2	0.751	0.585
FDBL	3	0.751	0.700
FDBL	4	0.634	0.700

PBL, partial bounding-box label; FSBL, full single bounding-box label; FDBL, full dual bounding-box label; EI, estrus-interaction. The seed = 0 row for each configuration comes from a standalone re-validation pass performed alongside the seeds 1–4 training campaign, using identical validation settings across all five seeds for internal consistency; it differs by ≤0.023 absolute from the originally reported seed = 0 matrix value used in Table 6/Table 10, which reflects ordinary run-to-run validation nondeterminism rather than a data discrepancy. Mean ± SD summaries are reported in Table 10 (main text).

Table S4. Effect of disabling mosaic and mixup augmentation, by annotation strategy (YOLO11n, held-out test split), together with the corrected YOLOv5n Refined-stage mixup values evaluated on the same test split, reported as a robustness check against Table 11 (main text, validation split).

Strategy	Mosaic/mixup	EI Recall	EI AP50	Overall mAP50
PBL	On (Refined)	0.531	0.560	0.763
PBL	Off	0.469	0.532	0.742
FSBL	On (Refined)	0.562	0.682	0.808
FSBL	Off	0.500	0.599	0.743
FDBL	On (Refined)	0.500	0.591	0.713
FDBL	Off	0.625	0.756	0.741
PBL, YOLOv5n Refined (mixup corrected)	On (Refined)	0.594	0.509	0.728
FSBL, YOLOv5n Refined (mixup corrected)	On (Refined)	0.625	0.684	0.791

PBL, partial bounding-box label; FSBL, full single bounding-box label; FDBL, full dual bounding-box label; EI, estrus-interaction. FDBL's mAP50 is averaged over four classes and is not directly comparable to the three-class mAP50 reported for PBL and FSBL. All values in this table, including the "On" baseline rows, are computed on the held-out test split (never used for checkpoint selection); the same configurations evaluated on the validation split (Table 11, main text) differ by up to approximately 0.23 absolute for individual cells, reflecting the different image content of the two splits rather than model instability, but show the same qualitative pattern across annotation strategies (FDBL benefits substantially from disabling mosaic/mixup; PBL and FSBL do not), indicating the ablation effect itself is not an artifact of the evaluation split.

Note on the FDBL + YOLO11n + Augmentation v1 test-split values reported in Section 3.13 of the main text (EI Recall 0.547, EI AP50 0.696, mAP50 0.723). These values were obtained from the only stored checkpoint for this configuration. Re-evaluated on the validation split, this checkpoint reproduced the EI Recall reported in Table 6 closely (0.691 vs. 0.700) but gave a higher EI AP50 (0.821 vs. 0.775), a difference larger than the 0.02 tolerance applied when checking the PBL and FSBL headline checkpoints. Two stored copies of the checkpoint gave identical results on both splits. The FDBL test-split values should therefore be read with this reproduction difference in mind.

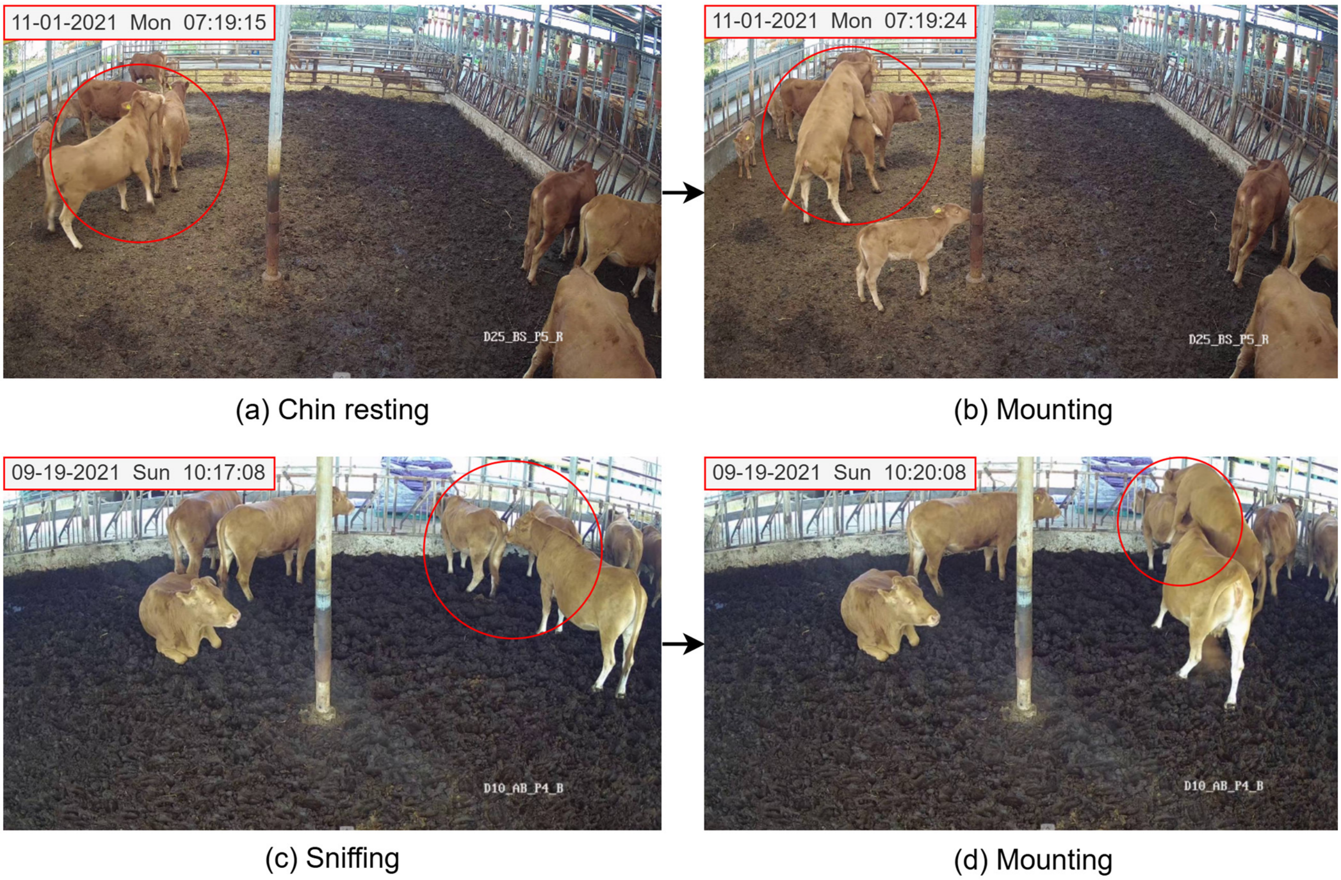

Figure S1. Representative frames showing chin-resting or sniffing occurring near mounting within the same camera scene.

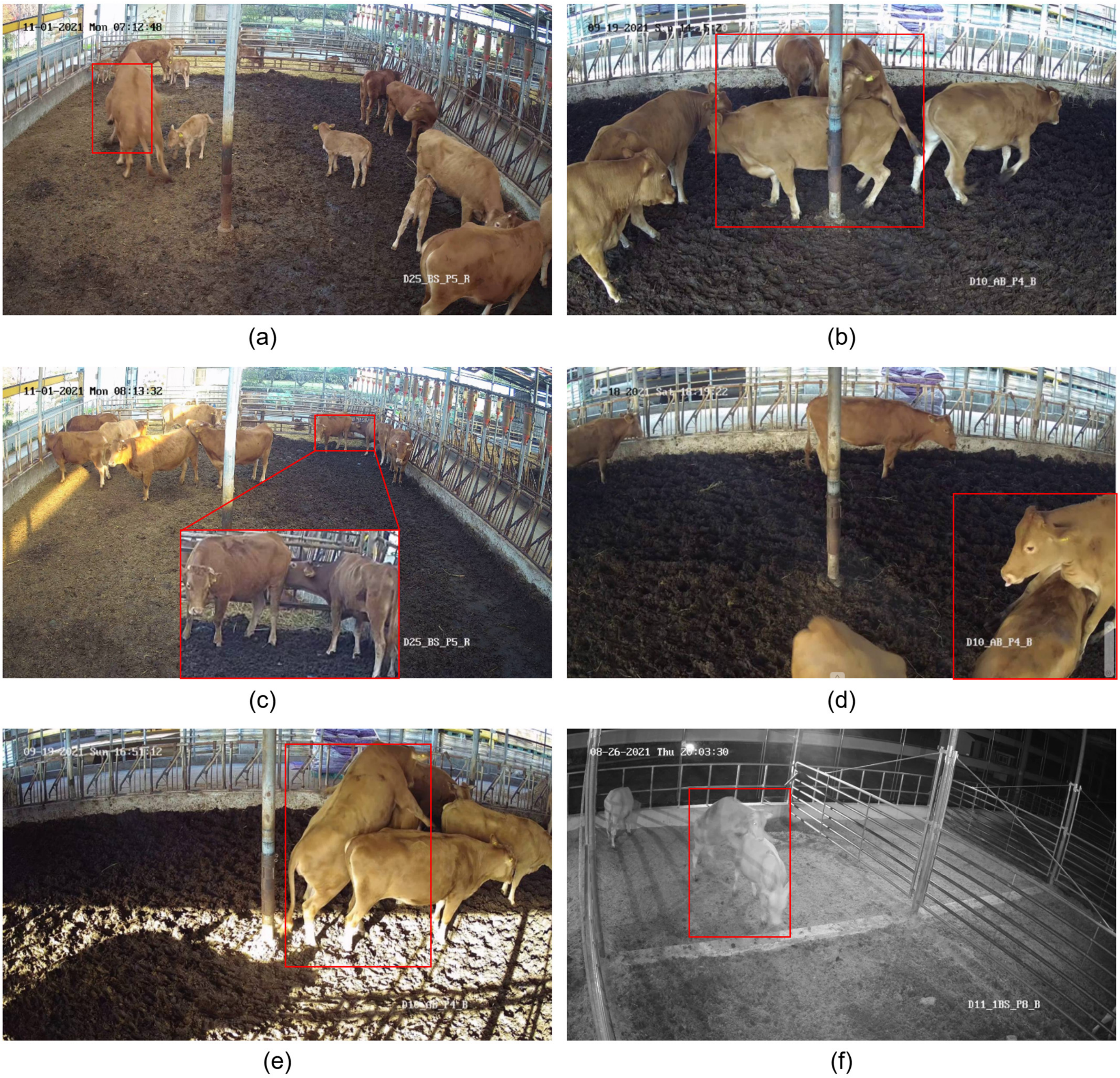

Figure S2. Representative difficult cases for estrus-interaction detection, including crowding, partial occlusion, overlapping animals, low light and ambiguous social contact.
